# Mid- to long-term outcomes of osteochondral lesions of the talus repair: a systematic review

**DOI:** 10.1186/s13018-025-06214-z

**Published:** 2025-10-14

**Authors:** Jimmy Wen, Burhaan Syed, Ihab Abed, Mouhamad Shehabat, Muzammil Akhtar, Daniel Razick, Arsh Alam, Christopher Kreuelen

**Affiliations:** 1 California Northstate University College of Medicine, 9700 W Taron Dr, Elk Grove, CA 95757 United States; 2 University of California, 1 Shields Ave, Davis, CA 95616 United States; 3 Department of Orthopaedic Surgery, University of California, Davis, 4860 Y St #1700, Sacramento, CA 95817 United States

**Keywords:** Osteochondral lesions of talus, OLT, BMS, MACI, AMIC, ACI, OATS

## Abstract

**Background:**

Osteochondral lesions of the talus (OLTs) involve damage to the articular cartilage and underlying bone, posing a therapeutic challenge due to the limited intrinsic healing capacity of cartilage. This review aims to provide mid- to long-term follow-ups for joint preservation procedures for OLTs through analysis of patient-reported outcomes (PROs), rates of return to activity/sport/work, and survival rates/complications.

**Methods:**

The guidelines by the Preferred Reporting Items for Systematic Reviews and Meta-Analyses (PRISMA) were followed to perform this systematic review in PubMed, Embase, and Cochrane Library for studies evaluating surgical treatment for OLTs at a minimum 5-year follow-up. Quality and risk of bias assessment were completed using the Methodological Index for Non-Randomized Studies criteria.

**Results:**

43 studies with a total of 2721 patients with an age range of 23.9 to 47.7 years, defect size of 0.9 to 29.1 cm^2^, and follow-up of 5 to 24.1 years were included. Survival rates, defined as no revision surgeries, at the latest follow-up, for bone marrow stimulation (8 studies), autologous chondrocyte implantation (6 studies), matrix-associated chondrocyte implantation (3 studies), autologous matrix-induced chondrogenesis (AMIC) (2 studies), OATS (7 studies), and bone marrow aspirate concentrate were 87.4 to 100%, 75 to 100%, 55.6 to 100%, 84.2 to 92.1%, 56.4 to 100%, and 90.1 to 95.3%. respectively. Survival rates for the other techniques, reported in separate studies, were biphasic bioresorbable scaffold (100%), matrix-induced stem cell transplantation (100%), AMIC plus peripheral blood concentrate (96.9%), microfracture plus platelet-rich plasma and hyaluronic acid (91.23%), autologous tibia osteoperiosteal graft (86.67%), arthroscopic lift-drill-fill-fix (66.7%), and particulates juvenile allograft cartilage implantation (53.5%). The majority of studies demonstrated significant PRO improvements (p < 0.05) at the latest follow-up.

**Conclusion:**

BMS remained the most commonly used technique (primarily used for smaller lesions) and produced survival rates comparable to other techniques included in this study. Most patients across all the techniques analyzed in this study had significant PRO improvements at the latest follow-up.

**Supplementary Information:**

The online version contains supplementary material available at 10.1186/s13018-025-06214-z.

## Introduction

Osteochondral lesions of the talus (OLTs) involve damage or deformity of the articular cartilage or underlying bone of the talus. These lesions are tough to treat due to the limited intrinsic healing capacity of cartilage, encapsulation of cells in the matrix, and an undifferentiated stem cell population, limiting their capacity to heal after injury [1]. A majority of OLTs result from impacts to the area, such as from repeated ankle sprains or trauma during sports. However, ischemia, repetitive microfractures, and genetic predisposition can also contribute to the development of an OLT [2]. Lesion location can be characterized as either lateral, medial, or central, although lateral and medial lesions are the most common [2, 3]. If left untreated, OLTs can have worsening outcomes, necessitating the need for swift diagnoses and treatment with good long-term outcomes.

Symptomatic OLTs will initially undergo conservative treatment, including rest, nonsteroidal anti-inflammatory drugs (NSAIDs), or immobilization. Additional conservative measures if the previous methods fail include corticosteroid and hyaluronic acid (HA) injections, platelet-rich plasma (PRP), and stem cell treatments [1, 2]. If conservative treatment fails, surgical intervention is required and can be broadly categorized into three options: repair (e.g., microfracture), replacement (e.g., autograft/allograft transplantation), and regeneration (e.g., cartilage implantation and chondrocyte implantation), mainly depending on lesion size [2].

Bone marrow stimulation (BMS) is the most frequently used treatment and is typically utilized for small (< 1.5 cm^2^) primary lesions and involves stimulating repair through shaving, debridement, drilling, or microfracturing of lesions to drive growth [4]. Osteochondral autologous/allogenic transplantation (OATS), on the other hand, aims to replace chondrocytes and repair the weight-bearing capabilities of the talus. It is generally used for larger lesions (> 1.5cm^2^) and can be used for both primary and secondary lesions [5]. Cell-based regenerative techniques such as Autologous Chondrocyte Implantation (ACI), matrix-associated ACI (MACI), and Autologous Matrix-induced Chondrogenesis (AMIC) are used in larger lesions (> 1.5cm^2^) and typically as salvage therapies or secondary lesions to induce chondrogenesis [5]. The advancement of new techniques and biomaterials for this category of treatment is rapidly evolving and can be a valuable option in the future of OLT treatment.

Despite an extensive array of procedures, there has been no study evaluating their long-term clinical efficacy and safety outcomes, especially with the continued introduction of new studies and techniques. By critically synthesizing OLT treatment outcomes in the literature at a minimum 5-year follow-up, this systematic review aims to evaluate the current state of knowledge regarding these techniques, identify potential gaps in the literature, and offer insights into future directions and clinical practice guidelines. We hypothesize that all techniques will significantly improve patient-reported outcomes (PROs) and have high survival rates.

## Methods

### Search strategy

The guidelines established by the Preferred Reporting Items for Systematic Reviews and Meta-Analyses (PRISMA), were utilized to perform a systematic search in three databases on June 19, 2025: PubMed, Embase, and Cochrane Library. The following search strategy were used to perform the systematic review: (((osteochondral) OR (chondral)) AND (((talar) OR (talus)) OR (ankle))) AND ((((((((follow-up) OR (outcome)) OR (mid-term)) OR (long-term)) OR (microfracture)) OR (chondrocyte transplantation)) OR (osteochondral transfer)) OR (matrix-induced)). The full search strategies for all databases can be found in Appendix S1. There were no limits set on the initial search.

The PICOT (Patient, Intervention, Comparison, Outcome, Time) method was utilized to guide our search strategy. The patient population is defined as adult patients over the age of 18 years. The intervention included the patient population undergoing surgical procedures for OLTs. Comparative studies with other treatment modalities were included. These included prospective/retrospective cohort studies, case–control studies, and case series with > 5 patients. Although randomized controlled trials (RCTs) were eligible, none met the minimum follow-up threshold. Outcomes assessed included PROs, return to activity, complications, and failures. Studies with a minimum mean 5-year follow-up were included. Inclusion criteria consisted of adult patients undergoing surgical treatment for OLTs. Exclusion criteria included case reports, cadaveric articles, reviews, technique studies, expert opinions, non-English studies, and studies without reported outcomes. No studies were excluded based on risk of bias. Two reviewers independently reviewed all the articles included in this study during title/abstract and full-text screening via a double-blinded dual-screening process on Covidence. If no consensus was reached, a third reviewer was consulted to assess final study inclusion or exclusion eligibility. Deduplication was done through Covidence’s automated algorithm, which removes duplicate studies by matching on key fields: title, authors, publication year, journal name, abstract, and DOI. Any remaining duplicates that were not screened through this algorithm were manually reviewed by two independent reviewers. A reference search was performed for all included studies to assess for additional possible studies to be added to this review. This protocol is registered under the PROSPERO CRD42023481375.

### Quality assessment

Two independent authors utilized the Methodological Index for Nonrandomized Studies (MINORS) criteria to evaluate the quality of the included studies [6]. MINORS scores ranged from 0 (not reported), 1 (reported but inadequate), or 2 (reported and adequate), with a maximum score of 16 for non-comparative studies and 24 for comparative studies. Any discrepancies were resolved by rigorous re-evaluation of the articles until an agreement was reached. Scores of 2 for seven or more Sects. (11 or more for comparative studies) were considered low risk of bias, five to six sections (nine to ten for comparative studies) were at moderate risk of bias, and four or less sections (eight or less for comparative studies) were high risk of bias. Given the uniformly high risk of bias, MINORS was used to contextualize the risk of bias rather than stratify results or pooled estimates.

GRADE (Grading of Recommendations, Assessment, Development and Evaluation) guidelines were used to evaluate the certainty of evidence. These guidelines assessed five domains: risk of bias, inconsistency, indirectness, imprecision, and publication bias. However, all included studies were non-RCTs and level IV evidence, resulting in downgrades for risk of bias and study design. Other downgrades were used for small sample sizes, variabilities in techniques, lesion sizes, and variable outcome reporting. Ratings were categorized as high, moderate, low, or very low.

### Data extraction and statistical analysis

Study variables evaluated in this systematic review include title, publication date, study year, number of patients/ankles, mean age ranges, mean follow-up time ranges, pre-and post-operative PROs, and rates of complications. Extracted data were collected and analyzed using Google Sheets (Google Drive; Google, Mountain View, CA). If applicable and available, descriptive statistics such as mean, percentage, standard deviations, and ranges were reported. Funding sources of included studies were not extracted. Missing data were not imputed or extrapolated.

“Survival” was defined as not requiring revision surgeries, as it was the most common and objectively verifiable outcome across the studies. Furthermore, PROs that are continuous and linear, which were originally reported on a scale of 0–10, were converted to a 0–100 scale to standardize the scores. Ordinal PROs that range from 0–10 were not converted to 0–100 as they are categorical and non-linear.

## Results

### Literature search

**Fig. 1:**
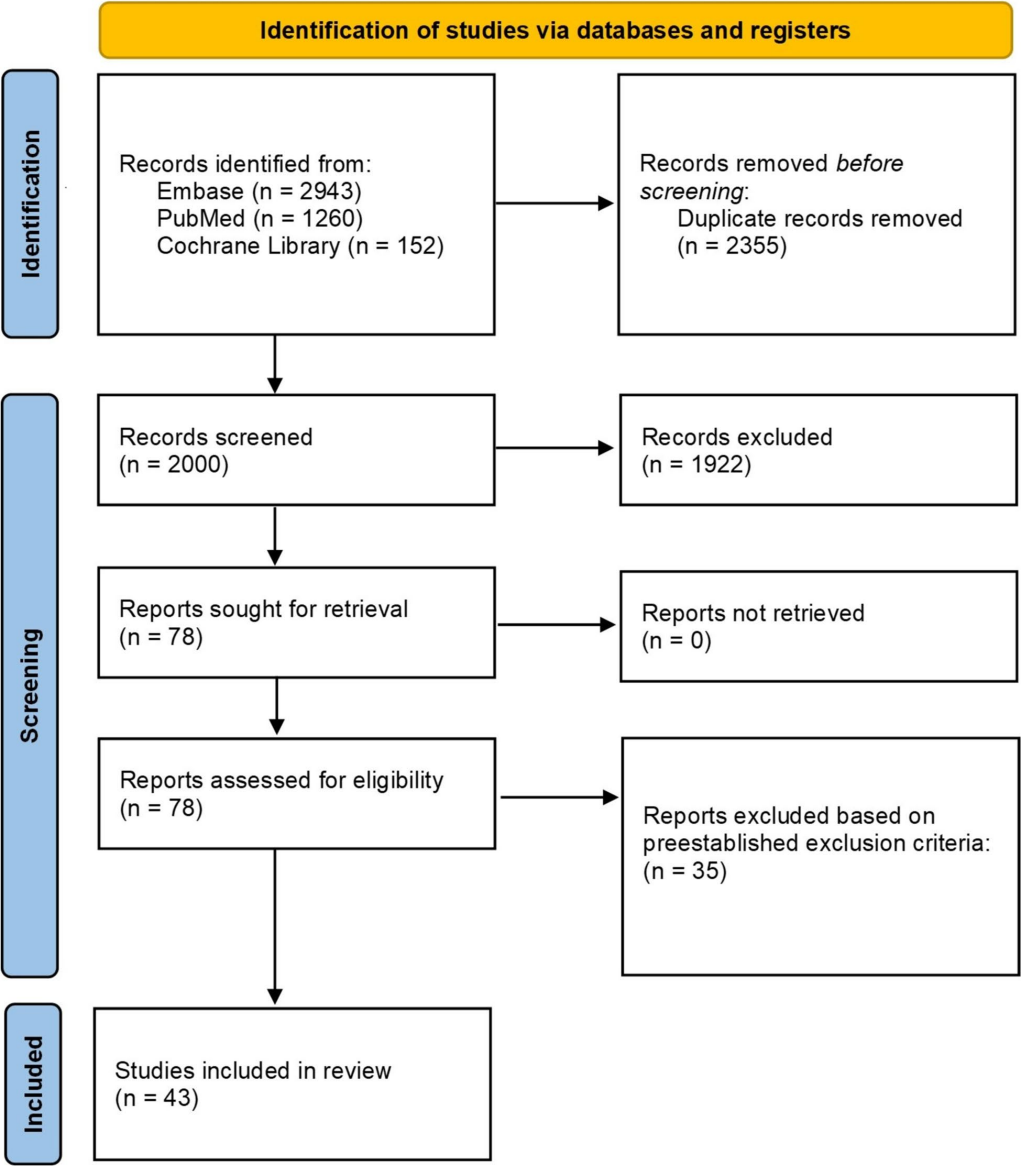
PRISMA diagram of included studies. Searches were conducted up until June 19, 2025 across PubMed, Embase and cochrane library

The initial search resulted in 2,720 studies through PubMed, Embase, and Cochrane Library. After removing 908 duplicates, 1,777 articles were screened by title and abstract for relevance, which yielded 54 articles. These articles were further screened with a full-text review, leaving 43articles to be included in this systematic review [7–49]. The article selection process is further detailed in Fig. 1.

### Study characteristics and demographics

Out of the 43 studies used, a total of 13 joint preservation techniques were evaluated in this review. There were a total of 2,721 patients across all studies (62.2% male; 37.8% female) with an age range of 23.9 to 47.7 years, defect size range of 0.9 to 29.1cm^2^, and follow-up time of 5 to 24.1 years. Across the 43 studies, there were seven ACI (156 patients) [8, 21, 22, 34, 43, 47, 48], eleven BMS (1,089 patients) [9, 10, 13, 29, 30, 35, 36, 40, 44, 45, 49], nine OATS (eight autograft/one fresh allograft, 288 patients) [12, 15, 18, 20, 25–27, 41, 42], three MACI (45 patients) [7, 28, 31], five AMIC (159 patients) [9, 14, 17, 20, 23, 24], two bone marrow aspirate concentrate scaffold (BMAC) (186 patients) [11, 46], and two autologous tibial osteoperiosteal grafts (103 patients) [32, 49]. There was also one of each study for biphasic bioresorbable scaffold (BBS) (12 patients) [16], matrix-associated stem cell transplantation (MAST) (120 patients) [37], AMIC plus peripheral blood concentrate (PBC) (129 patients) [38], microfracture (MFx) plus PRP and HA (365 patients) [19], particulated juvenile allograft cartilage implantation (PJCAT) (13 patients) [33], and arthroscopic Lift-Drill-Fill-Fix technique (LDFF) (18 patients) [39]. The demographic data and study characteristics across the studies can be found in Table S1.

### Methodological quality and risk of bias

Out of 43 studies, six studies were comparative [9, 20, 25, 30, 41, 49], with a MINORS score ranging from 18 to 21. For the other 37 non-comparative studies, the MINORS score ranged from 12 to 16. The risk of bias for five comparative studies was determined to be moderate, and one was high, while for non-comparative studies, 21 were determined to be moderate and 16 were low (Table S2).

### Outcome scores for joint preservation procedures

42 studies reported at least one PRO for each of the joint preservation techniques. For the BMS technique, mean preoperative and postoperative ranges were reported for the American Orthopaedic Foot & Ankle Society score (AOFAS) (52.8 and 73 to 85.5 and 90.8) in 5studies [10, 30, 35, 36, 49]. For OATS, mean preoperative and postoperative scores for AOFAS were reported for three studies (47.7 to 55.4 and 80.6 to 92.1) [15, 20, 42]. For ACI, ranges for mean preoperative and postoperative scores are as follows: AOFAS in seven studies (40.4 to 89.4 and 86.2 to 94.7) [8, 21, 22, 34, 43, 47]. For MACI, the mean ranges for AOFAS in three studies were 60.2 to 70.1 and 78.3 to 95.3 [7, 21, 23]. Autologous tibial osteoperiosteal graft technique reported preoperative and postoperative AOFAS in only two studies (49.1 to 56.6 and 82.8 to 88.7) [32, 49]. Outcome values and significance for all studies can be found summarized in Table S3. For studies that reported AOFAS, all reported a significant increase from before the procedure, regardless of technique.

In Anders et al., with the MACI technique, age, gender, body mass index (BMI), defect localization, defect size, and duration of symptoms did not significantly affect the results of AOFAS, Visual Analogue Scale (VAS), and magnetic resonance observation of cartilage repair tissue score (MOCART) [7]. Lenz et al. also found a very weak relationship between MOCART and other outcomes, such as Foot and Ankle Ability Measure (FAAM) and AOFAS, indicating that for MACI, the MOCART score did not correlate with clinical outcomes [31]. AMIC procedures had similar findings, whereas in a study done by Gottschalk et al., gender, smoking status, previous surgical interventions, etiology, defect location, or symptom duration did not have a significant effect on the Foot Function Index-Disability (FFI-D) [23]. They found a significant positive relationship between pre-operative FFI-D with age and BMI, along with postoperative FFI-D and lesion size.

With ACI, Giannini et al. found significantly better recovery if the patient was under 40 (p = 0.08) [22]. A significant difference between previously treated patients versus never before treated patients for cartilage repair, with patients who have already been treated reporting lower outcomes (p = 0.05). With OATS, del'Escalopier et al. found that there were significant factors for poorer outcomes, such as work-accident or occupational disease status (p = 0.048), frontal laxity (p = 0.027 in three cases), preoperative osteoarthritis (p = 0.022), lesion size in the sagittal plane (p = 0.031), and high BMI (p = 0.02) [15]. Lesion location or the number of plugs showed no significant correlation.

### Outcome scores by procedure type

Given the variability in procedures reported across the studies, a subgroup analysis was conducted based on procedure type. These include cell-based therapies (ACI, MACI, AMIC, MAST, BMAC), scaffold-based therapies (BBS), BMS (BMS, MFx plus PRP and HA), and osteochondral grafting (OATS, osteoperiosteal graft, LDFF, PJCAT). Cell-based therapies had the highest post-operative range for AOFAS at 78.3 to 95.3, while the osteochondral grafting group had the lowest range at 75 to 92.1. Results are summarized in Table 1and GRADE certainty can be found in Table S4.

**Table 1:** Outcomes Ranges by Joint Preservation Techniques

Technique	Procedures	Number of Studies (n)	Patient-Reported Outcome (n studies that reported)	Pre-operative Ranges	Post-operative Ranges	Follow-up Range (months)	Lesion Size Range (cm^2^)	GRADE Certainity
Cell-Based	ACI, MACI, AMIC, MAST, BMAC	19	AOFAS (15 studies)	40.4 to 89.4 (13 studies)	78.3 to 95.3 (15 studies)	60 to 289.3	0.3 to 6.9	Low
			Tegner activity level (4 studies)	1 to 2.4 (3 studies)	3 to 5.7 (4 studies)			
			VAS (6 studies)	57 to 87 (4 studies)	9 to 33 (6 studies)			
Scaffold-Based	BBS	1	AOFAS (1 study)	47.2 ± 10.7	84.4 ± 8	78 to 104.4	NR	Very Low
Bone Marrow Stimulation	BMS, MFx plus PRP and HA	12	AOFAS (4 studies)	58.2 to 58.7 (2 studies)	82.8 to 90 (4 studies)	60 to 183.6	0.1 to 1.8	Low
Osteochondral Grafting	OATS, PJCAT, osteoperiosteal graft, LDFF	13	AOFAS (6 studies)	47.7 to 56.6 (5 studies)	75.3 to 92.1 (6 studies)	63.5 to 166.1	0.5 to 3.6	Low
			FAOS (7 studies)	39.6 to 52.3 (5 studies)	75.3 to 91.2 (7 studies)			

### Return to activity, work, and sports

For return to activity (RTA), Anders et al. reported one patient who exceeded the pre-operative activity level, with an overall rate of 18/22 (81.8%) for MACI [7]. Vannini et al. reported 69/101 (68.3%) RTA for the BMAC technique [46]. For return to work (RTW), using the BBS, DiCave et al. stated that the average time to return to work was 8 and 12 weeks for sedentary and standing/walking work, respectively [16]. With BMS, Lambers et al. reported that of the 58/60 (96.7%) who RTW, one worked less due to ankle problems, seven worked less due to other reasons, and four worked more due to a decrease in ankle problems [29]. Using ACI, Winkler et al. stated that, although 31/35 (88.6%) returned to work, three came back with minor limitations, six with major limitations, and four remained disabled [48].

The return to sports (RTS) rate range for ACI (three studies) was 86.2% to 100% [8, 22, 48]. Giannini et al. reported that among the 29 patients who played a sport, 20 RTS at the same level, three RTS at a lower level, two changed to a non-contact sport, and four gave up sports [22]. It was also reported that four professional soccer players had the best clinical and functional outcomes and were able to RTS at the same level. Winkler et al. reported that 6/35 (17.1%) had no limitations, 8/35 (22.9%) had minor limitations, and 21/35 (60%) had major limitations when RTS [48]. With BMS, the range of RTS was 74% to 90% for the four studies that reported RTS. Lambers et al. (60 patients) report an RTS of 32 to pre-injury level, with 20 not at pre-injury level due to ankle problems, and 8 not at pre-injury levels due to circumstances not involving the ankle [29]. Using OATS, Keszég et al. (24 patients) reported that 16 patients had an RTS at the same level, with 7 at a lower level, and an overall RTS of 23/24 (95.8%) [26]. Kim et al. and Fiske et al. demonstrated RTS rates of 23/28 (82.1%) and 18/34 (52.9%), respectively, using OATS [18, 27]. RTA/RTW/RTS information is summarized in Tables S5 and S6.

### Complications and revisions

For BMS, four studies commented on complications, ranging from 0 to 8% [30, 35, 36, 44]. For ACI, four studies reported a range of 0 to 15% [8, 21, 22, 34, 43]. For MACI, two studies reported a range of 0 to 19% [7, 28]. Eight OATS studies reported a range of 0% to 59% [12, 15, 20, 25–27, 41, 42]. One study with PJCAT reported 7.7% [33]. Studies with BBS [16], MAST [37], AMIC plus PBC [38], and LDFF [39] reported 0% complications.

Revision rates were reported in six ACI studies [8, 21, 22, 34, 43, 48], three MACI studies [7, 28, 31], eight BMS studies [10, 13, 29, 35, 36, 40, 44, 45], two BMAC studies [11, 46], two AMIC studies [17, 24], and seven OATS studies [12, 15, 18, 20, 27, 41, 42]. Rates were reported in one study each for BBS [16], MFx plus PRP and HA [19], autologous tibial osteoperiosteal grafts [31], MAST [37], AMIC plus PBC [38], PJCAT [33], and arthroscopic LDFF [39]. Highest survival rates, defined as no revision surgeries, belonged to the BBS technique (100%), followed by MAST (97.5%), AMIC plus PBC (96.9%), BMAC (90.1 to 95.3%), MFx plus PRP and HA (91.2%), BMS (range: 87.4 to 100%), AMIC (range: 84.2 to 92.1%), MACI (range: 55.6 to 100%), OATS (range: 56.4 to 100%), autologous tibial osteoperiosteal grafts (86.7%), ACI (range: 75 to 100%), LDFF (66.7%), and finally PJCAT (53.8%). Reoperations, revisions, complications, and survival rates can be found in Table S4 for all individual studies.

## Discussion

This systematic review assessed the outcomes of OLT joint preservation techniques at a minimum 5-year follow-up through analysis of PROs, functional outcomes, and complications/survival rates. The main findings in this systematic review were that 1) BMS was the most commonly used technique (used primarily for smaller lesions), appearing in eight studies, and produced comparable survival rates (92.72%) compared to all other techniques included in this study (53.8% to 100%). (2) Most studies (93.5%) demonstrated significant PRO improvements at the latest follow-up. (3) Of the 19 studies that reported complications, OATS reported the highest range, from 3.7% to 59%. While several studies reported on RTA/RTW/RTS across multiple techniques, these were not stratified based on lesion sizes. Previous studies have suggested that larger lesion sizes, such as over 150 mm^2^ for BMS, are associated with delayed RTS [2]. However, due to the included studies not categorizing these outcomes based on lesion sizes, definite conclusions across the various surgical methodologies could to be made.

Although these findings provide insight into minimum 5-year outcomes of surgical treatments for OLT, they must be interpreted cautiously due to the considerable heterogeneity across studies. Key sources of heterogeneity included wide lesion size ranges (0.9 to 29.1cm^2^), and characteristics such as deep or cystic lesions, as these can influence the surgical treatment choice as well as affect outcomes. Concomitant or previous procedures, such as previous cartilage procedures, were variably reported and not controlled for, which introduces further confounding variables. Baseline demographics such as age, sex, and activity level were not often stratified into sub-groups for outcome reporting. Thus, these variables limited comparability and the conduction of a meta-analysis. Future studies that control and stratify these variables will allow for greater comparability across studies. Additionally, the majority of studies relied mainly on PROs such as AOFAS and VAS, with varying usage of objective measures like MOCART or other radiologic measures. Although PROs are clinically relevant, the objective outcomes can help reveal structural healing, graft incorporation, durability, and long-term joint stability. This underscores the need for further standardized protocols in future research to integrate both subjective and objective measures.

### Conservative treatment

Conservative treatment for OLTs is generally considered first-line treatment but is associated with a 45% success rate and 46% pooled conversion rate to surgery [50]. Additionally, patients with lower levels of pain, activity limitations, and higher preoperative PROs are more appropriate candidates for conservative treatment. A 2022 systematic review found that conservative treatment was the most commonly used intervention for skeletally immature patients, suggesting that younger patients may benefit more from conservative treatment due to their greater healing potential compared to adults [51]. Other conservative modalities that have been investigated are HA and PRP. In a quasirandomized controlled trial, Mei-Dan et al. compared the usage of HA or PRP in a series of 3 consecutive intra-articular injections and followed for 28 weeks [52]. At final follow-up, the mean Ankle-Hindfoot Scale (AHFS), VAS, and subjective global function scores all improved significantly compared to baseline, although PRP also had a significant improvement in these parameters when compared to HA [52]. These modalities can be considered as first-line treatments in the short term, but the long-term outcomes for larger or more complex lesions are still uncertain.

It is important to distinguish between talus bone bruises versus OLTs as their management is different [53]. OLTs can appear like a bone bruise, as bone edema and contusions can be seen in the acute setting. However, OLTs have a subchondral bone plate injury with or without cartilage disruption. Bone bruises follow a benign course, with symptom resolution ranging from six to eight weeks, while OLTs are less predictable and require rehabilitation, conservative treatment modalities, or surgeries if the lesion is large, chronic, or unstable [53].

### Commonly-used joint-preservation techniques

Several joint-preserving techniques have been developed to address OLTs. A 2021 network meta-analysis including BMS, OATS, AMIC, and MACI with a median follow-up of 47.8 months found that AMIC demonstrated the highest AOFAS scores (standardized mean difference: 11.27), lowest VAS scores (standardized mean difference: −2.26), and the lowest rates of revisions and failures (log odds ratio: 0.94 for both). On the other hand, OATS produced the highest rates of revisions (log odds ratio: 4.60) and failures (log odds ratio: 3.48) [54].

BMS is the most commonly used procedure, primarily for small lesions, usually less than 1.5 cm^2^ [55]. Similarly, Anwander et al. found that BMS was the most commonly used technique, but mainly for primary lesions with a mean size less than 1 cm^2^ [56]. They also reported that patients with lower preoperative AOFAS scores, larger lesion sizes, and more symptomatic OLTs had the greatest increase in clinical scores. A pooled analysis from 2018 found that BMS yielded an 82% success rate, defined as an AOFAS of over 80 [51]. This was also seen in a retrospective analysis at a mean follow-up of 13.9 years with an AOFAS score of 82.76 ± 11.65 [35]. However, results are less favorable in larger and more extensive lesions [57]. Interestingly, BMS studies in the knee tend to deteriorate over time, but our findings for BMS in the OLT suggest stable mid- to long-term survival rates. This may be a reflection of the anatomical and biomechanical differences in the ankle compared to the knee. For example, the ankle experiences less shear forces during its main movements, and the incidence of osteoarthritis is relatively low and usually a result of post-trauma, compared to the knee, which develops osteoarthritis idiopathically [2].

In terms of OATS, the pooled success rates (AOFAS over 80) for autografts were found to be 77% for primary lesions and 90% for secondary lesions. For allografts, the pooled success rates ranged from 20 to 100% for primary lesions and 55% for secondary lesions [5]. A comparison between allograft versus autografts across 1174 patietns with a mean follow-up of 46.5 months found signficant improvements for autografts in AOFAS (mean difference: 4.8, p = 0.04) and MOCART (mean difference: 10.5, p = 0.04) scores while demonstrating lower rates of revisions (odds ratio: 7.2, p < 0.0001) and failures (odds ratio: 5.1, p < 0.0001) compared to allografts [58]. The authors suggest that allografts should primarily be used only if a suitable donor site is not available, with the higher rates of failures being attributed to possible immunogenic responses that are similar to those in allogenic organ transplants [58]. Although OATS provides great results as a salvage therapy or for larger lesions, donor site morbidity (autograft) and the need for malleolar osteotomy both increase the risk for complications.

PJCAT is a one-step procedure that utilizes prepackaged allografts from young donors under the age of 13 years with viable chondrocytes and hyaline cartilage. It theoretically obviates the risk of donor site morbidity and is primarily used for difficult-to-treat OLTs (moderate-sized lesions or salvage therapy after MFx). Juvenile cartilage produces higher levels of extracellular matrix proteins, type II collagen, and contains a higher density in articular cartilage compared to adult variants [33]. However, it appears that despite the improved clinical outcome scores, there does not seem to be observed repair of subchondral bone or lamina, resulting in a lesser likelihood of full restoration of normal hyaline cartilage [59].

For MACI and AMIC, the pooled treatment success for primary lesions ranges from 56 to 100% and secondary lesions range from 57% to 72 [5]. The disadvantages of ACI and MACI are that they require a two-stage operation; however, the latter provides an advantage over the former by avoiding a periosteal graft, providing a more even cell distribution, and having the ability to perform the procedure arthroscopically [60].

On the other hand, AMIC is a one-step procedure that utilizes bone marrow-derived mesenchymal stem cells (MSCs) derived from the subchondral bone and provides a quicker recovery with reduced overall costs [61]. A 2022 systematic review of 778 patients with a mean follow-up of 37.4 months found that VAS (p < 0.001), AOFAS (p < 0.001), FFI (p = 0.02), and MOCART (p = 0.03) improved significantly, but not Tegner score (p = 0.08) [62]. Notably, the revision and failure rates were 7.8% and 6.2%, respectively, but the authors acknowledge that the included studies did not clearly report complications and failures, potentially underestimating the true rates [62]. Additionally, AMICs use as a revision procedure shows potential. Migliorini et al. performed a revision AMIC for a previously failed AMIC at a mean follow-up of 34.9 years and a larger mean defect size of 3.2 cm^2^, found mean AOFAS, VAS, and Tegner scores of 58.8 ± 20.6, 41 ± 31, and 3.5 ± 1.6 [63]. However, the authors did not collect the preoperative PROs, preventing a comparison of the improvement from baseline to the latest follow-up. The MOCART score significantly improved (22.1 ± 13.7 to 42.3 ± 27.9, p = 0.04), but 30% (8/27) underwent further revision with a chondral procedure [63]. However, Becher et al. compared MFx and AMIC and found no significant inter-group differences in PROs [9]. Though MFx/BMS is commonly used for smaller lesions, the addition of a scaffold with AMIC can be considered for primary and revision cases with lesion sizes greater than 1 cm^2^, as well as cases where bone grafting might be required [9].

MAST is a modification of AMIC in combination with stem cell transplantation and is indicated for unstable, fragmented, or missing cartilage. Richter et al. compared their 2-year follow-up to their 5-year follow-up and found that at 2 years, approximately half of their patients had visible lesions under MRI, but these were not found at 5 years [37]. AMIC plus PBC had equivalent findings compared to MAST. Richter et al. suggested that the lack of difference between BMAC or PBC addition to AMIC suggests that AMIC alone would provide the same results [30]. Recent evidence in the literature supports the usage of scaffolds and regenerative techniques for cystic and larger lesions [57].

### Emerging or less commonly used joint-preservation techniques

BBS is a novel biodegradable scaffold composed of polylactide-co-glycolide, calcium sulfate, and polyglycolide fibers, aiming to be a scaffold for native marrow and extracellular matrix growth [16]. Its bioresorbable nature avoids donor site morbidity associated with OATS or the usage of foreign material. However, if not implanted flush to the articular surface, they can lead to increased damage and erosion to articular structures or lead to synovitis secondary to synovial membrane irritation [16]. Autologous osteoperiosteal cylinder grafts are a one-stage procedure that involves obtaining grafts from the medial tibia and transplanting the graft using the same incision, decreasing donor-site morbidity associated with OATS or two-step procedures such as ACI/MACI [32]. Li et al. utilized this technique for large cystic OLTs, with a low recurrence rate of cysts at 11.76% compared to other techniques, ranging from 46.4% to 76.9% [32]. The sustained long-term results of their technique are due to the graft’s periosteum and subchondral bone, which show sustained positive results over time [32]. LDDF is a fixation technique where subchondral drilling and autologous bone grafting are performed, and thus can be categorized as an intra-articular non-union repair. It is indicated for primary OLTs > 1 cm and > 0.3 cm thickness. Fixation’s advantage over other techniques is its preservation of native hyaline cartilage, restoration of the talar dome, immediate stabilization, and facilitating subchondral bone restoration [39]. BMAC is also a one-step procedure and contains MSCs, precursor stem cells, and accessory cells that express growth factors to prompt angiogenesis and vasculogenesis [46].

### Complications

A 2023 meta-analysis of complications across 178 studies (6,962 OLTs) reported a total of 225 complications (5%), which is similar to the findings in this study (6.4%) [64]. BMS (67 studies/2926 lesions), LDFF (8 studies/179 lesions), cartilage implantation including MACI and AMIC (22 studies/479 lesions), osteochondral autograft transplantation (61 studies/1639 lesions), osteochondral allograft transplantation (15 studies/288 lesions) had a complication rate of 4%, 3%, 5%, 8%, and 8%, respectively [64]. Complication grading was assessed utilizing the Modified Clavien-Dindo-Sink Complication Classification System for Orthopedic Surgery. Grades I, II, and III had rates of 4%, 3%, and 3%, respectively, with no significant differences found between treatment modalities. There were also no Grade IV or Grade V found [64]. The most common complication noted for BMS, LDFF, and cartilage implantation was nerve injury (dysesthesia, neuralgia, paresthesia, or altered sensation of temporary or unknown duration). For OATS autograft and allograft, it was donor site morbidity (41%) and deep venous thrombosis (27%), respectively [64]. It is important to note that operative time and the experience of the surgeon are important factors that can affect the complication rate of these procedures [65, 66]. Additionally, the definition of complications varies and leads to high heterogeneity in the reporting of complications in the literature. Treatment failures (graft complications), persistent pain, no improvement of function, and removal of implanted hardware may not be explicitly considered as complications. Thus, as suggested by Hollander et al., we agree that an established definition for complications should be agreed upon and would be useful in standardizing this term and treatment complication rates [64]. Although complications may be low in some studies, the long-term clinical impact can vary. Minor complications such as transient swelling, stiffness, pain, or nerve neuropraxia are usually self-limited and do not affect the long-term function of the joint. However, more severe complications such as persistent pain, graft failures, or those that require revision procedures more significantly impact quality of life, especially if revisions are required, as that increases recovery time and healthcare costs.

### Implications

The findings of this systematic review are clinically relevant by providing a concise overview of the clinical results of the various available joint preservation techniques for OLT. Thus, this information can be used by clinicians to guide their treatment choices and provide up-to-date information for patients on expected outcomes after their procedure, thereby better facilitating the shared decision-making process. However, treatment must be personalized and evidence-based based on differing patient and lesion characteristics (depth, location, size, cystic) before final treatment selection. For example, although OLTs are a three-dimensional defect, grading typically follows a two-dimensional approach. Saxena et al. graded OLTs used an algorithm that encompasses volume, location, and integrity of the subchondral plate, to classify small (less than 125 mm^3^), medium (125–1500 mm^3^), and large (greater than 1500 mm^3^) [67]. Using this algorithm, the authors utilized MFx, retrograde drilling, OATS, and an open or arthroscopic approach with promising results, having 3.4% (7/204) requiring a revision surgery [67].

Other important factors in the shared decision-making process include complication characteristics, cost, and efficacy. Differing baseline patient characteristics, including age, activity level, history of prior procedures, and concomitant ankle pathologies (fractures/instability/impingement), can affect the clinical outcomes as well. Patients with higher baseline activity levels or athletes may benefit greater from techniques that optimize the quality and stability of the cartilage, although these may be more technically complex. Thus, future studies should focus on standardizing patient demographics and conducting high-quality RCTs to better elucidate the outcomes of these surgical techniques. The optimal treatment for OLTs is still debated, and there is no clear consensus for one technique over another. Shared decision-making should consider both anatomical and lifestyle factors, underscoring the importance of a personalized surgical approach for OLTs.

This systematic review has several strengths to note. First, the analysis of PROs, functional outcomes, and survival rates provides a comprehensive review of the current literature on several joint preservation techniques for OLTs. PROs and associated p-values were reported to indicate whether there were significant improvements pre- to post-operation. Second, all included studies had a minimum follow-up of 5 years, thus providing the aforementioned outcomes at a mid to long-term follow-up.

### Limitations

However, the findings of this study must also be understood within the context of its limitations. First, there was notable heterogeneity in the included studies regarding baseline patient characteristics, study designs, concomitant or previous procedures, lesion sizes, and outcomes reported. This was also seen in the heterogeneity in “survival” definitions across the studies. In this study, we defined survival as the absence of revision surgery; however, some of the included studies used different definitions, such as pain resolution or graft integrity. These differences in reporting can cause overestimation and variation within the reported survival rates. Based on GRADE assessment, the certainty of the evidence was low, and we refrained from overinterpreting the findings. Future studies should standardize how outcomes are defined and reported, reducing heterogeneity, allowing direct comparison across studies, and facilitating a more definitive synthesis of treatment outcomes of OLT. Future studies should also stratify patients based on lesion characteristics, including size, depth, and the presence of subchondral cysts, as this may lead to differential responses to different interventions, allowing for personalized treatment decisions. Second, only English studies were included, which may introduce language bias and missing relevant literature published in other languages. Third, the majority of studies were non-comparative in design. The included studies were level of evidence IV, which limits the overall strength of the findings and prevents a meta-analysis of pooled data. Higher methodological quality studies (RCTs) with larger sample sizes and follow-up periods may influence the results and better approximate actual clinical outcome scores, PROs, and complication rates. It must also be noted that several studies had a low number of patients, and future studies with larger cohorts may adjust the findings in this review. Fourth, subjective outcomes (PROs) were reported; there was a paucity of objective outcomes, such as radiological findings. These objective outcome measures can complement PRO findings and provide a more robust evaluation of treatment efficacy, recovery, and satisfaction. Fifth, there were several procedures reported in only one study, which limits the validity and interpretation of those findings.

## Conclusion

BMS remained the most commonly used technique (primarily for smaller lesions) and produced comparable survival rates compared to all other techniques included in this study. Novel techniques that were included demonstrated variable survival rates but may not be adequately reflected due to being minimally performed in single studies. The majority of patients across all the techniques demonstrated significant PRO improvements at the latest follow-up.

## Supplementary Information

Supplementary Material 1. 

Supplementary Material 2.

Supplementary Material 3.

Supplementary Material 4.

Supplementary Material 5.

Supplementary Material 6.

Supplementary Material 7.

## Data Availability

The datasets used and/or analyzed in the current study are available upon reasonable request. Please contact J.W. to request data from the study.

## Abbreviations

OLT: Osteochondral lesions of the talus
NSAIDS: Non-steroidal anti-inflammatory drugs
HA: Hyaluronic acid
PRP: Platelet-rich plasma
BMS: Bone marrow stimulation
OATS: Osteochondral autologous/Allogenic transplantation
ACI: Autologous chondrocyte implantation
MACI: Matrix-associated ACI
AMIC: Autologous matrix-induced chondrogenesis
PRO: Patient-reported outcomes
PRISMA: Preferred reporting items for systematic reviews and meta-analyses
PICOT: Patient, intervention, comparison, outcome, time
RCT: Randomized controlled trials
MINORS: Methodological index for nonrandomized studies
GRADE: (Grading of Recommendations, Assessment, Development and Evaluation)
BBS: Biphasic bioresorbable scaffold
MAST: Matrix-associated stem cell transplantation
PBC: Peripheral blood concentrate
MFx: Microfracture
BMAC: Bone marrow aspirate concentrate scaffold
PJCAT: Particulates juvenile allograft cartilage implantation
LDFF: Lift-drill-fill-fix
AOFAS: American orthopaedic foot & ankle society score
VAS: Visual analogue scale
BMI: Body mass index
MOCART: Magnetic resonance observation of cartilage repair tissue
FAAM: Foot and ankle ability measure
FFI-D: Foot function index-disability
RTA: Return to activity
RTW: Return to work
RTS: Return to sport
MSCs: Mesenchymal stem cells

## References

[1] Badekas T, Takvorian M, Souras N. Treatment principles for osteochondral lesions in foot and ankle. Int Orthop. 2013;37(9):1697–706. 10.1007/s00264-013-2076-1. 23982639 10.1007/s00264-013-2076-1 PMC3764304

[2] Lan T, McCarthy HS, Hulme CH, Wright KT, Makwana N. The management of talar osteochondral lesions - current concepts. Journal of Arthroscopy and Joint Surgery. 2021;8(3):231–7. 10.1016/j.jajs.2021.04.002. 34337329 10.1016/j.jajs.2021.04.002 PMC8312263

[3] Deng E, Gao L, Shi W, et al. Both magnetic resonance imaging and computed tomography are reliable and valid in evaluating cystic osteochondral lesions of the talus. Orthop J Sports Med. 2020;8(9): 2325967120946697. 10.1177/2325967120946697. 32995345 10.1177/2325967120946697 PMC7503027

[4] Dahmen J, Lambers KTA, Reilingh ML, van Bergen CJA, Stufkens SAS, Kerkhoffs GMMJ. No superior treatment for primary osteochondral defects of the talus. Knee Surg Sports Traumatol Arthrosc. 2018;26(7):2142–57. 10.1007/s00167-017-4616-5. 28656457 10.1007/s00167-017-4616-5 PMC6061466

[5] Rikken QGH, Kerkhoffs GMMJ. Osteochondral lesions of the talus: an individualized treatment paradigm from the Amsterdam perspective. Foot Ankle Clin. 2021;26(1):121–36. 10.1016/j.fcl.2020.10.002. 33487235 10.1016/j.fcl.2020.10.002

[6] Slim K, Nini E, Forestier D, Kwiatkowski F, Panis Y, Chipponi J. Methodological index for non-randomized studies (minors): development and validation of a new instrument: Methodological index for non-randomized studies. ANZ J Surg. 2003;73(9):712–6. 10.1046/j.1445-2197.2003.02748.x. 12956787 10.1046/j.1445-2197.2003.02748.x

[7] Anders S, Goetz J, Schubert T, Grifka J, Schaumburger J. Treatment of deep articular talus lesions by matrix associated autologous chondrocyte implantation–results at five years. Int Orthop. 2012;36(11):2279–85. 10.1007/s00264-012-1635-1. 22885840 10.1007/s00264-012-1635-1 PMC3479272

[8] Baums MH, Heidrich G, Schultz W, Steckel H, Kahl E, Klinger HM. Autologous chondrocyte transplantation for treating cartilage defects of the talus. J Bone Joint Surg Am. 2006;88(2):303–8. 10.2106/JBJS.E.00033. 16452741 10.2106/JBJS.E.00033

[9] Becher C, Malahias MA, Ali MM, Maffulli N, Thermann H. Arthroscopic microfracture vs. arthroscopic autologous matrix-induced chondrogenesis for the treatment of articular cartilage defects of the talus. Knee Surg Sports Traumatol Arthrosc. 2019;27(9):2731–6. 10.1007/s00167-018-5278-7. 30392029 10.1007/s00167-018-5278-7

[10] Becher C, Zühlke D, Plaas C, et al. T2-mapping at 3 T after microfracture in the treatment of osteochondral defects of the talus at an average follow-up of 8 years. Knee Surg Sports Traumatol Arthrosc. 2015;23(8):2406–12. 10.1007/s00167-014-2913-9. 24562698 10.1007/s00167-014-2913-9

[11] Berveglieri L, Vannini F, Ramponi L, et al. The influence of cell and platelet number on clinical outcomes provided by a one-step scaffold transplantation with bone marrow concentrate for the treatment of osteochondral lesions of the talus. Foot Ankle Surg. 2025. 10.1016/j.fas.2025.01.014. 39984338 10.1016/j.fas.2025.01.014

[12] Butler JJ, Robert G, Dahmen J, et al. Outcomes following autologous osteochondral transplantation for osteochondral lesions of the talus at 10-year follow-up: A retrospective review. Cartilage. Published online 2024:19476035241293268. 10.1177/19476035241293268 10.1177/19476035241293268 PMC11556656 39469788

[13] Corr D, Raikin J, O’Neil J, Raikin S. Long-term outcomes of microfracture for treatment of osteochondral lesions of the talus. Foot Ankle Int. 2021;42(7):833–40. 10.1177/1071100721995427. 33719632 10.1177/1071100721995427

[14] Deiss L, Walther M, Pfahl K, et al. Long-term results after autologous matrix-induced chondrogenesis for osteochondral lesions of the talus: A 10-year cohort study. Cartilage. Published online 2024:19476035241301896. 10.1177/19476035241301896 10.1177/19476035241301896 PMC11635787 39665329

[15] de l’Escalopier N, Amouyel T, Mainard D, et al. Long-term outcome for repair of osteochondral lesions of the talus by osteochondral autograft: A series of 56 Mosaicplasties®. Orthop Traumatol Surg Res. 2021;107(8S):103075. 10.1016/j.otsr.2021.103075 10.1016/j.otsr.2021.103075 34563735

[16] Di Cave E, Versari P, Sciarretta F, Luzon D, Marcellini L. Biphasic bioresorbable scaffold (TruFit Plug®) for the treatment of osteochondral lesions of talus: 6- to 8-year follow-up. Foot Edinb. 2017;33:48–52. 10.1016/j.foot.2017.05.005. 29126043 10.1016/j.foot.2017.05.005

[17] Efrima B, Barbero A, Maccario C, et al. Significant clinical improvement after arthroscopic autologous matrix-induced chondrogenesis for osteochondral lesions of the talus: A 5-year follow-up. Cartilage. Published online 2024:19476035241240341. 10.1177/19476035241240341 10.1177/19476035241240341 PMC11569665 38554040

[18] Fiske JW, Dalal AH, McCauley JC, Bugbee WD. Participation in sports or recreational activities after osteochondral allograft transplantation of the talus. Am J Sports Med. 2024;52(5):1258–64. 10.1177/03635465241234890. 38523479 10.1177/03635465241234890

[19] Fu S, Yang K, Li X, et al. Radiographic and clinical outcomes after arthroscopic microfracture for osteochondral lesions of the talus: 5-year results in 355 consecutive ankles. Orthop J Sports Med. 2022;10(10): 23259671221128772. 10.1177/23259671221128772. 36263313 10.1177/23259671221128772 PMC9575450

[20] Gedikbas M, Ozturk T, Asci M, Erpala F, Sobay U, Güneş T. Comparison of autologous matrix-induced chondrogenesis and mosaicplasty in the treatment of osteochondral defects of the talus. Acta Orthop Traumatol Turc. 2024;58(5):301–7. 10.5152/j.aott.2024.23001. 39560972 10.5152/j.aott.2024.23001 PMC11583942

[21] Giannini S, Battaglia M, Buda R, Cavallo M, Ruffilli A, Vannini F. Surgical treatment of osteochondral lesions of the talus by open-field autologous chondrocyte implantation: a 10-year follow-up clinical and magnetic resonance imaging T2-mapping evaluation: A 10-year follow-up clinical and magnetic resonance imaging T2-mapping evaluation. Am J Sports Med. 2009;37 Suppl 1(1_suppl):112S-8S. 10.1177/0363546509349928 10.1177/0363546509349928 19934440

[22] Giannini S, Buda R, Ruffilli A, et al. Arthroscopic autologous chondrocyte implantation in the ankle joint. Knee Surg Sports Traumatol Arthrosc. 2014;22(6):1311–9. 10.1007/s00167-013-2640-7. 23996105 10.1007/s00167-013-2640-7

[23] Gottschalk O, Altenberger S, Baumbach S, et al. Functional medium-term results after autologous matrix-induced chondrogenesis for osteochondral lesions of the talus: a 5-year prospective cohort study. J Foot Ankle Surg. 2017;56(5):930–6. 10.1053/j.jfas.2017.05.002. 28647522 10.1053/j.jfas.2017.05.002

[24] Götze C, Nieder C, Felder H, Peterlein CD, Migliorini F. AMIC for traumatic focal osteochondral defect of the talar shoulder: a 5 years follow-up prospective cohort study. BMC Musculoskelet Disord. 2021;22(1):638. 10.1186/s12891-021-04506-z. 34303367 10.1186/s12891-021-04506-z PMC8310607

[25] Haleem AM, Ross KA, Smyth NA, et al. Double-plug autologous osteochondral transplantation shows equal functional outcomes compared with single-plug procedures in lesions of the Talar dome: A minimum 5-year clinical follow-up: A minimum 5-year clinical follow-up. Am J Sports Med. 2014;42(8):1888–95. 10.1177/0363546514535068. 24948585 10.1177/0363546514535068

[26] Keszég M, Pánics G, Gulácsi G, Tóth G, Hangody L. Long-term outcomes of talus osteochondral autologous transplantation in soccer players: 24 mosaicplasty with more than 10 years of follow-up. Journal of Cartilage & Joint Preservation. 2022;2(2): 100061. 10.1016/j.jcjp.2022.100061.

[27] Kim SH, Cho BK, Choi SM, Kim SH. Clinical and radiologic outcomes following autologous osteochondral transplantation for lateral osteochondral lesions of the talus. Foot Ankle Int. 2025;46(2):182–91. 10.1177/10711007241308576. 39825769 10.1177/10711007241308576

[28] Kreulen C, Giza E, Walton J, Sullivan M. Seven-year follow-up of matrix-induced autologous implantation in talus articular defects. Foot Ankle Spec. 2017;11(2):1938640017713614. 10.1177/1938640017713614. 10.1177/1938640017713614 28587484

[29] Lambers KTA, Dahmen J, Altink JN, Reilingh ML, van Bergen CJA, Kerkhoffs GMMJ. Bone marrow stimulation for talar osteochondral lesions at long-term follow-up shows a high sports participation though a decrease in clinical outcomes over time. Knee Surg Sports Traumatol Arthrosc. 2021;29(5):1562–9. 10.1007/s00167-020-06250-8. 32918555 10.1007/s00167-020-06250-8 PMC8038982

[30] Lee GW, Lee MS, Kim JE, Lee KB. The effect of smoking on the outcomes of arthroscopic microfracture for osteochondral lesions of the talus. PLoS One. 2025;20(4): e0321894. 10.1371/journal.pone.0321894. 40261898 10.1371/journal.pone.0321894 PMC12013919

[31] Lenz CG, Tan S, Carey AL, Ang K, Schneider T. Matrix-induced autologous chondrocyte implantation (MACI) grafting for osteochondral lesions of the talus. Foot Ankle Int. 2020;41(9):1099–105. 10.1177/1071100720935110. 32639169 10.1177/1071100720935110

[32] Li Y, Tang Y, Wang Z, et al. Improved results from medium- and long-term outcomes of autogenous osteoperiosteal grafting for large cystic lesions of the talus. Arthroscopy. 2024;40(5):1613–22. 10.1016/j.arthro.2023.09.022. 37821015 10.1016/j.arthro.2023.09.022

[33] Manzi J, Arzani A, Hamula MJ, Manchanda K, Dhanaraj D, Chapman CB. Long-term patient-reported outcome measures following particulated juvenile allograft cartilage implantation for treatment of difficult osteochondral lesions of the talus. Foot Ankle Int. 2021;42(11):1399–409. 10.1177/10711007211014173. 34112022 10.1177/10711007211014173

[34] Pagliazzi G, Vannini F, Battaglia M, Ramponi L, Buda R. Autologous chondrocyte implantation for talar osteochondral lesions: comparison between 5-year follow-up magnetic resonance imaging findings and 7-year follow-up clinical results. J Foot Ankle Surg. 2018;57(2):221–5. 10.1053/j.jfas.2017.05.013. 29146220 10.1053/j.jfas.2017.05.013

[35] Park JH, Park KH, Cho JY, Han SH, Lee JW. Bone marrow stimulation for osteochondral lesions of the talus: Are clinical outcomes maintained 10 years later? Am J Sports Med. 2021;49(5):1220–6. 10.1177/0363546521992471. 33661712 10.1177/0363546521992471

[36] Polat G, Erşen A, Erdil ME, Kızılkurt T, Kılıçoğlu Ö, Aşık M. Long-term results of microfracture in the treatment of talus osteochondral lesions. Knee Surg Sports Traumatol Arthrosc. 2016;24(4):1299–303. 10.1007/s00167-016-3990-8. 26831855 10.1007/s00167-016-3990-8

[37] Richter M, Zech S. Matrix-associated stem cell transplantation (MAST) in chondral lesions at the ankle as part of a complex surgical approach- 5-year-follow-up in 100 patients. Foot Ankle Surg. 2019;25(3):264–71. 10.1016/j.fas.2017.11.004. 29409182 10.1016/j.fas.2017.11.004

[38] Richter M, Zech S, Meissner S, Naef I. Autologous matrix induced chondrogenesis plus peripheral blood concentrate (AMIC+PBC) in chondral lesions at the ankle as part of a complex surgical approach - 5-year follow-up. Foot Ankle Surg. 2022;28(8):1321–6. 10.1016/j.fas.2022.06.015. 35803836 10.1016/j.fas.2022.06.015

[39] Rikken QGH, Altink JN, Dahmen J, Lambers KTA, Stufkens SAS, Kerkhoffs GMMJ. Sustained clinical success at 7-year follow-up after arthroscopic lift-drill-fill-fix (LDFF) of primary osteochondral lesions of the talus. Knee Surg Sports Traumatol Arthrosc. 2023;31(5):1978–85. 10.1007/s00167-022-07243-5. 36602563 10.1007/s00167-022-07243-5 PMC10090019

[40] Rikken QGH, Aalders MB, Dahmen J, Sierevelt IN, Stufkens SAS, Kerkhoffs GMMJ. Ten-year survival rate of 82% in 262 cases of arthroscopic bone marrow stimulation for osteochondral lesions of the talus. J Bone Joint Surg Am. 2024;106(14):1268–76. 10.2106/JBJS.23.01186. 38728384 10.2106/JBJS.23.01186 PMC11608585

[41] Shimozono Y, Yasui Y, Hurley ET, Paugh RA, Deyer TW, Kennedy JG. Concentrated bone marrow aspirate may decrease postoperative cyst occurrence rate in autologous osteochondral transplantation for osteochondral lesions of the talus. Arthroscopy. 2019;35(1):99–105. 10.1016/j.arthro.2018.06.047. 30424945 10.1016/j.arthro.2018.06.047

[42] Suh JW, Kwon JH, Lee DH, Jung JU, Park HW. Outcomes of osteochondral autologous transplantation with ipsilateral lateral Talar autograft for medial osteochondral lesions of the talus. Clin Orthop Surg. 2024;16(4):620–7. 10.4055/cios23327. 39092295 10.4055/cios23327 PMC11262937

[43] Toker B, Erden T, Çetinkaya S, Dikmen G, Özden VE, Taşer Ö. Long-term results of osteochondral autograft transplantation of the talus with a novel groove malleolar osteotomy technique. Jt Dis Relat Surg. 2020;31(3):509–15. 10.5606/ehc.2020.75231. 32962583 10.5606/ehc.2020.75231 PMC7607963

[44] van Bergen CJA, Kox LS, Maas M, Sierevelt IN, Kerkhoffs GMMJ, van Dijk CN. Arthroscopic treatment of osteochondral defects of the talus: outcomes at eight to twenty years of follow-up. J Bone Joint Surg Am. 2013;95(6):519–25. 10.2106/JBJS.L.00675. 23515986 10.2106/JBJS.L.00675

[45] van Eekeren ICM, van Bergen CJA, Sierevelt IN, Reilingh ML, van Dijk CN. Return to sports after arthroscopic debridement and bone marrow stimulation of osteochondral talar defects: a 5- to 24-year follow-up study. Knee Surg Sports Traumatol Arthrosc. 2016;24(4):1311–5. 10.1007/s00167-016-3992-6. 26846661 10.1007/s00167-016-3992-6 PMC4823315

[46] Vannini F, Berveglieri L, Boffa A, et al. Hyaluronic scaffold transplantation with bone marrow concentrate for the treatment of osteochondral lesions of the talus: durable results up to a minimum of 10 years. Knee Surg Sports Traumatol Arthrosc. 2023;31(10):4551–8. 10.1007/s00167-023-07490-0. 37328684 10.1007/s00167-023-07490-0

[47] Viglione V, Berveglieri L, Filardo G, et al. Autologous chondrocyte implantation for the treatment of osteochondral lesions of the talus: What happens after 20 years? Foot Ankle Surg. 2024;30(7):546–51. 10.1016/j.fas.2024.04.007. 38653636 10.1016/j.fas.2024.04.007

[48] Winkler PW, Geyer S, Walzl D, et al. Favorable long-term clinical and radiologic outcomes with high survivorship after autologous osteochondral transplantation of the talus. Knee Surg Sports Traumatol Arthrosc. 2023;31(6):2166–73. 10.1007/s00167-022-07237-3. 36394584 10.1007/s00167-022-07237-3 PMC10183420

[49] Yang S, Shao Q, Zhu Y, et al. Surgical treatment for medium-sized cystic osteochondral lesions of the talus: Autologous osteoperiosteal transplantation provides better clinical outcomes than bone marrow stimulation when cysts are deeper than 6 mm. Knee Surg Sports Traumatol Arthrosc. 2025;33(2):750–9. 10.1002/ksa.12388. 39091253 10.1002/ksa.12388

[50] Buck TMF, Lauf K, Dahmen J, Altink JN, Stufkens SAS, Kerkhoffs GMMJ. Non-operative management for osteochondral lesions of the talus: a systematic review of treatment modalities, clinical- and radiological outcomes. Knee Surg Sports Traumatol Arthrosc. 2023;31(8):3517–27. 10.1007/s00167-023-07408-w. 37062042 10.1007/s00167-023-07408-w PMC10356662

[51] Dahmen J, Steman JAH, Buck TMF, et al. Treatment of osteochondral lesions of the talus in the skeletally immature population: a systematic review. J Pediatr Orthop. 2022;42(8):e852–60. 10.1097/BPO.0000000000002175. 35605211 10.1097/BPO.0000000000002175 PMC9351694

[52] Mei-Dan O, Carmont MR, Laver L, Mann G, Maffulli N, Nyska M. Platelet-rich plasma or hyaluronate in the management of osteochondral lesions of the talus. Am J Sports Med. 2012;40(3):534–41. 10.1177/0363546511431238. 22253252 10.1177/0363546511431238

[53] McCollum GA, Calder JDF, Longo UG, et al. Talus osteochondral bruises and defects: diagnosis and differentiation. Foot Ankle Clin. 2013;18(1):35–47. 10.1016/j.fcl.2012.12.002. 23465947 10.1016/j.fcl.2012.12.002

[54] Migliorini F, Maffulli N, Schenker H, et al. Surgical management of focal chondral defects of the talus: a bayesian network meta-analysis. Am J Sports Med. 2022;50(10):2853–9. 10.1177/03635465211029642. 34543085 10.1177/03635465211029642 PMC9354066

[55] Zengerink M, Struijs PAA, Tol JL, van Dijk CN. Treatment of osteochondral lesions of the talus: a systematic review. Knee Surg Sports Traumatol Arthrosc. 2010;18(2):238–46. 10.1007/s00167-009-0942-6. 19859695 10.1007/s00167-009-0942-6 PMC2809940

[56] Anwander H, Vetter P, Kurze C, Farn CJ, Krause FG. Evidence for operative treatment of talar osteochondral lesions: a systematic review. EFORT Open Rev. 2022;7(7):460–9. 10.1530/EOR-21-0101. 35900197 10.1530/EOR-21-0101 PMC9297053

[57] Walther M, Gottschalk O, Aurich M. Operative management of osteochondral lesions of the talus: 2024 recommendations of the working group “clinical tissue regeneration” of the German Society of Orthopedics and Traumatology (DGOU). EFORT Open Rev. 2024;9(3):217–34. 10.1530/EOR-23-0075. 38457916 10.1530/EOR-23-0075 PMC10958247

[58] Migliorini F, Maffulli N, Baroncini A, et al. Allograft versus autograft osteochondral transplant for chondral defects of the talus: systematic review and meta-analysis. Am J Sports Med. 2022;50(12):3447–55. 10.1177/03635465211037349. 34554880 10.1177/03635465211037349 PMC9527449

[59] Aldawsari K, Alrabai HM, Sayed A, Alrashidi Y. Role of particulated juvenile cartilage allograft transplantation in osteochondral lesions of the talus: a systematic review. Foot Ankle Surg. 2021;27(1):10–4. 10.1016/j.fas.2020.02.011. 32169329 10.1016/j.fas.2020.02.011

[60] Tan H, Li A, Qiu X, et al. Operative treatments for osteochondral lesions of the talus in adults: A systematic review and meta-analysis: A systematic review and meta-analysis. Medicine (Baltimore). 2021;100(25): e26330. 10.1097/MD.0000000000026330. 34160396 10.1097/MD.0000000000026330 PMC8238309

[61] Migliorini F, Maffulli N, Baroncini A, Knobe M, Tingart M, Eschweiler J. Matrix-induced autologous chondrocyte implantation versus autologous matrix-induced chondrogenesis for chondral defects of the talus: a systematic review. Br Med Bull. 2021;138(1):144–54. 10.1093/bmb/ldab008. 33940611 10.1093/bmb/ldab008

[62] Migliorini F, Maffulli N, Bell A, Hildebrand F, Weber CD, Lichte P. Autologous matrix-induced chondrogenesis (AMIC) for osteochondral defects of the talus: a systematic review. Life (Basel). 2022;12(11): 1738. 10.3390/life12111738. 36362893 10.3390/life12111738 PMC9693539

[63] Migliorini F, Schenker H, Maffulli N, et al. Autologous matrix induced chondrogenesis (AMIC) as revision procedure for failed AMIC in recurrent symptomatic osteochondral defects of the talus. Sci Rep. 2022;12(1):16244. 10.1038/s41598-022-20641-6. 36171261 10.1038/s41598-022-20641-6 PMC9518950

[64] Hollander JJ, Dahmen J, Emanuel KS, Stufkens SAS, Kennedy JG, Kerkhoffs GMMJ. The frequency and severity of complications in surgical treatment of osteochondral lesions of the talus: a systematic review and meta-analysis of 6,962 lesions. Cartilage. 2023;14(2):180–97. 10.1177/19476035231154746. 37144397 10.1177/19476035231154746 PMC10416205

[65] Cheng H, Chen BPH, Soleas IM, Ferko NC, Cameron CG, Hinoul P. Prolonged operative duration increases risk of surgical site infections: a systematic review. Surg Infect (Larchmt). 2017;18(6):722–35. 10.1089/sur.2017.089. 28832271 10.1089/sur.2017.089 PMC5685201

[66] Birkmeyer JD, Finks JF, O’Reilly A, et al. Surgical skill and complication rates after bariatric surgery. N Engl J Med. 2013;369(15):1434–42. 10.1056/NEJMsa1300625. 24106936 10.1056/NEJMsa1300625

[67] Saxena A, Maffulli N, Jin A, Isa E, Jaswal J, Allen R. Outcomes of talar osteochondral and transchondral lesions using an algorithmic approach based on size, location, and subchondral plate integrity: a 10-year study on 204 lesions. J Foot Ankle Surg. 2022;61(3):442–7. 10.1053/j.jfas.2021.06.011. 35249808 10.1053/j.jfas.2021.06.011

