# Correction: Mid- to long-term outcomes of osteochondral lesions of the talus repair: a systematic review

**DOI:** 10.1186/s13018-026-06692-9

**Published:** 2026-02-26

**Authors:** Jimmy Wen, Burhaan Syed, Ihab Abed, Mouhamad Shehabat, Muzammil Akhtar, Daniel Razick, Arsh Alam, Christopher D. Kreulen

**Affiliations:** 1 California Northstate University College of Medicine, 9700 W Taron Dr, Elk Grove, CA 95757 USA; 2 University of California, 1 Shields Ave, Davis, CA 95616 USA; 3 Department of Orthopaedic Surgery, University of California, Davis, 4860 Y St #1700, Sacramento, CA 95817 USA

**Correction to: Journal of Orthopaedic Surgery and Research (2025) 20:892**

10.1186/s13018-025-06214-z

In this article [1], the co-author’s name Christopher D. Kreulen was incorrectly written as Christopher Kreuelen

Incorrect Name:

Christopher Kreuelen

Correct Name:

Christopher D. Kreulen
