# Supplementary material for: Mid- to long-term outcomes of osteochondral lesions of the talus repair: a systematic review
Source: J Orthop Surg Res. 2025 Oct 14;20:892. doi: 10.1186/s13018-025-06214-z (PMC12522747; doi:10.1186/s13018-025-06214-z)
Supplement: Supplementary file 1 — Supplementary Material 1. [file 13018_2025_6214_MOESM1_ESM.docx]

Appendix S1: Search Strategy

PubMed (06/19/2025): 1260 results

(((osteochondral) OR (chondral)) AND (((talar) OR (talus)) OR (ankle))) AND ((((((((follow-up) OR (outcome)) OR (mid-term)) OR (long-term)) OR (microfracture)) OR (chondrocyte transplantation)) OR (osteochondral transfer)) OR (matrix-induced))

Embase (06/19/2025): 2943 results

(((osteochondral) OR (chondral)) AND (((talar) OR (talus)) OR (ankle))) AND ((((((((follow-up) OR (outcome)) OR (mid-term)) OR (long-term)) OR (microfracture)) OR (chondrocyte transplantation)) OR (osteochondral transfer)) OR (matrix-induced))

Cochrane Library (06/19/2025): 152 results

(((osteochondral) OR (chondral)) AND (((talar) OR (talus)) OR (ankle))) AND ((((((((follow-up) OR (outcome)) OR (mid-term)) OR (long-term)) OR (microfracture)) OR (chondrocyte transplantation)) OR (osteochondral transfer)) OR (matrix-induced))

*No explosions (e.g. *) or explosions (e.g. MeSH) were used for the search without filters or limits set on the search. The search used keyword-based Boolean search strings only*
