# Supplementary material for: Mid- to long-term outcomes of osteochondral lesions of the talus repair: a systematic review
Source: J Orthop Surg Res. 2025 Oct 14;20:892. doi: 10.1186/s13018-025-06214-z (PMC12522747; doi:10.1186/s13018-025-06214-z)
Supplement: Supplementary file 2 — Supplementary Material 2. [file 13018_2025_6214_MOESM2_ESM.docx]

**Table S1: Study Characteristics and Patient Demographics**

| Author | Journal | Study Year | Study Design | LOE | Number of Patients (M/F) | Mean Age (Years) | Mean Follow-Up (Months) | Mean Lesion size (cm²) |  |
| --- | --- | --- | --- | --- | --- | --- | --- | --- | --- |
| Anders 2012 | International Orthopaedics | 2003-2007 | Prospective case series | 4 | 17/5 | 23.9 (15-43) | 63.5 ±7.4 | 1.94 (range 1–6) |  |
| Baums 2006 | The Journal of Bone & Joint Surgery | - | Prospective case series | 4 | 5/7 | 29.7 | 63 | 2.3 |  |
| Becher 2015 | Knee Surgery, Sports Traumatology, Arthroscopy | - | Prospective cohort | 4 | 7/8 | 37 ± 17 | 94.8 ± 26.4 | Group A: 1.11 ± 52 (range 0.37–1.89) |  |
| Becher 2019 | Knee Surgery, Sports Traumatology, Arthroscopy | - | Retrospective case series | 4 | 14/18 | Group A (AMIC) 33.3±9.3 group B (MFx) 32.4±12.5 | Group A 67.2 ± 6 Group B 68.4 ± 8.4 | Group B: 1.06±47 (range 0.52–1.98) |  |
| Berveglieri 2025 | Foot and Ankle Surgery | - | Prospective case series | 4 | 53/32 | 32.3 ± 10.6 | 150.9 | 0.87 ± 30 (range 0.37–1.89) |  |
| Butler 2024 | Cartilage | January 2006 - October 2013 | Retrospective cohort | 4 | 27/12 | 36.3 ± 13.3 | 138.9 ± 16.9 | 2.7 ± 1.6 |  |
| Corr 2021 | Foot & Ankle International | 2007-2009 | Retrospective case series | 4 | 24/21 | 37.6 | 139.4 | 1.22 ± 64.1 |  |
| Deiss 2024 | Cartilage | June 2010 - November 2022 | Prospective case series | 4 | 12/6 | 39 ± 15 | 120 | 0.74 (range, 0.20-2.70) |  |
| del'Escalopier 2021 | Orthopaedics & Traumatology: Surgery & Research | - | Retrospective case series | 4 | 37/19 | 34 (18-60) | 102 ( 60-240) | 1.4 ± 0.9 (range 0.2-4) |  |
| DiCave 2017 | The Foot | March 2007-April 2009 | Retrospective case series | 4 | 9/3 | 38.6 (22 -57) | 90 (78-104.4) | 0.9 (range 0.25-3) |  |
| Efrima 2024 | Cartilage | January 2013 - January 2017 | Retrospective case series | 4 | 32/31 | 37 | 80 | NR |  |
| Fiske 2024 | The American Journal of Sports Medicine | - | Retrospective case series | 4 | 17/17 | 36.1 (20.5-57.7) | 110.4 | 1.40 (1.30-1.75) |  |
| Fu 2022 | Orthopaedic Journal of Sports Medicine | May 1, 2011, to May 31, 2015 | Prospective cohort | 4 | 269/86 | 28.4 ± 6.3 | 60 | 3.6 (range, 1-7.2) |  |
| Gedikbas 2024 | Acta Orthopaedica et Traumatologica Turcica | 2010-2020 | Retrospective cohort | 4 | Group I: 11/17  Group II: 12/10 | Group I: 39.7 ± 13.5  Group II: 43.95 ± 12.5 | 69.9 | 0.95 ± .32 |  |
| Giannini 2009 | The American Journal of Sports Medicine | 1997 and 1999 | Retrospective case series | 4 | 5/5 | 25.8 ± 6.4 | 119 ± 6.5 | Group I: 1.6 ± 0.73 |  |
| Giannini 2014 | Ankle | 2001 to 2006 | Retrospective case series | 4 | 29/17 | 31.4 ± 7.6 | 87.2 ± 14.5 | Group II: 1.29 ± 0.5 |  |
| Gottschalk 2017 | Journal of Foot and Ankle Surgery | - | Prospective cohort | 4 | 13/8 | 37 ± 15 | 60 | 3.1 (range 2.2-4.3) |  |
| Götze 2021 | BMC Musculoskeletal Disorders | - | Prospective cohort | 4 | 9/10 | 47.3 ± 13.2 | 66.2 ± 11.6 | 1.6 (range 1.0–4.0) |  |
| Haleem 2014 | The American Journal of Sports Medicine | 2003 and 2007 | Case-control | 4 | dp aot: 7/7  sp aot: 17/11 | dp AOT: 42.79 ± 11.9  sp AOT: 44.14 ± 11 | dp aot: 93.04 ± 15.08  sp aot: 85.29 ± 17.55 | 1.4 ± 0.9 (range 0.2 - 4.0) |  |
| Keszég 2022 | Journal of Cartilage & Joint Preservation | 1997 and 2011 | Retrospective case series | 4 | 11/13 | 24.33 ± 7.46 (13-37) | 166.1 ± 43.68 | 6.9±2.2 |  |
| Kim 2025 | American Orthopaedic Foot & Ankle Society | August 2013  - March 2021 | Retrospective case series | 4 | 19/9 | 35.4 ± 9.5 | 68.5 ± 26.9 | 2.08±54 |  |
| Kreulen 2018 | Foot & Ankle Specialist | - | Prospective case series | 4 | 4/5 | 45.8 ± 13.7 | 84 | 2.92± .46 |  |
| Lambers 2021 | Knee Surgery, Sports Traumatology, Arthroscopy | 2009 to 2014 | Prospective case series | 4 | NR | 39 ± 8.5 | 76.8 ± 13.2 | 1.34 ± 0.39 (1-2) |  |
| Lee 2025 | PLOS One | January 2005 - December 2021 | Retrospective cohort | 4 | Smoker group:58/1  Non Smoker Group: 108/83 | Smoker Group: 35.9 ± 13.1  Non Smoker Group: 38.8 ± 15.6 | Smoker Group: 86.8 ± 55.5  Non Smoker Group: 82.6 ± 51.0 | 1.71±0.7 |  |
| Lenz 2020 | Foot & Ankle International | August 2003-February 2006 | Retrospective case series | 4 | 4/11 | 36 ± 10 | 144 ± 12 | 1.29 (range 0.60-2.60) |  |
| Li 2023 | Arthroscopy: The Journal of Arthroscopic and Related Surgery | - | Retrospective case series | 4 | 46/29 | 41.23 ± 10.34 | 75.6 | NR |  |
| Manzi 2021 | Foot & Ankle International | November 2010 and May 2012 | Prospective cohort | 4 | 8/5 | 46.5 ± 11.8 | 97.8 ± 26.6 | Smoker Group: 0.74 ± 0.27 |  |
| Pagliazzi 2018 | The Journal of Foot & Ankle Surgery | - | Retrospective case series | 4 | 6/14 | 35 ± 8 | 87.2 ± 14.5 | (0.26–1.46) |  |
| Park 2021 | The American Journal of Sports Medicine | January 2001 - December 2008 (surgery dates) | Retrospective case series | 4 | 128/74 | 39.17 ± 14.44 | 166.8 ± 24.62 | Non Smoker Group: 0.70 ± 0.30 (0.20–1.48) |  |
| Polat 2016 | European Society of Sports Traumatology | 1996 - 2009 (surgery dates) | Retrospective case series | 4 | 48/34 | 35.9 ± 13.4 | 121.3 ± 35.1 | 2.04 |  |
| Richter 2019 | Foot and Ankle Surgery | April 2009 - May 2012 (surgery dates) | Prospective case series | 4 | 74/46 | 35 | 60.2 | 1.51 ± 0.53 |  |
| Richter 2022 | Foot and Ankle Surgery | July 2016 - May 2017 | Prospective case series | 4 | 77/52 | 35.6 | 60 (56–64) | (range 70-260) |  |
| Rikken 2023 | Knee Surgery, Sports Traumatology, Arthroscopy | - | Prospective case series | 4 | 9/9 | 24.2 ± 15.2 | 82.9 ± 9.3 | 0.27 ± 0.10 |  |
| Rikken 2024 | The Journal of Bone & Joint Surgery | - | Retrospective cohort | 4 | 162/100 | 32.3 ± 11.7 | 183.6 | 1.05 (range, 0.19-3.22) |  |
| Shimozono 2019 | The Journal of Arthroscopic & Related Surgery. | 2004-2008 | Retrospective cohort | 4 | AOT alone: 17/9  AOT with CBMA:  21/7 | AOT alone: 33.6 ± 12.3  AOT with CBMA:  36.0 ± 15.3 | 92.8 ± 13.8 | 1.7 ± 0.7 (0.25–5) |  |
| Suh 2024 | Clinics in Orthopedic Surgery | 2011-2022 | Retrospective case series | 4 | 7/4 | 47.7  (23–68) | 64.7 (14–137) | 1.7 (0.8–6) |  |
| Toker 2020 | Joint Diseases and  Related Surgery | 2002-2008 | Retrospective case series | 4 | 11/9 | 33.5 ± 11 | 143.5 | 1.8 (range 0.6–4) |  |
| vanBergen 2013 | The Journal of Bone and Joint Surgery | 1988-2000 | Retrospective case series | 4 | 30/20 | 32 ± 10 | 141 ± 34 | 1.30±0.07 |  |
| vanEekeren 2016 | Knee Surgery Sports Traumatology Arthroscopy | - | Retrospective case series | 4 | 61/32 | 32.6 ± 9.5 | 118 (46–271) | 0.73 ± 49.8 |  |
| Vannini 2023 | Knee Surgery, Sports Traumatology, Arthroscopy | 2006 to 2012 | Prospective case series | 4 | 64/37 | 32.9 ± 10.9 | 151.5 ± 18.4 | 1.03±0.42 |  |
| Viglione 2024 | Foot and Ankle Surgery | December 1997 - October 2002 | Prospective case series | 4 | 5/4 | 25.2 ± 6.3 | 289.3 ± 21.2 | 0.51 (range, 0.34–0.71) |  |
| Winkler 2023 | Knee Surgery Sports Traumatology Arthroscopy | 1997-2003 | Retrospective case series | 4 | 19/26 | 32.2 ± 8.9 | 229.2 ± 16.8 | 1.20 |  |
| Yang 2025 | Knee Surgery Sports Traumatology Arthroscopy | 2014-2019 | Retrospective cohort | 4 | Grafted Group: 16/12  BMS Group: 17/10 | Grafted Group: 43.1 ± 11.7  BMS Group:  41.5 ± 12.8 | 63.5 ± 13.9 | 0.11 ± 0.04 |  |
| LOE: level of evidence, AMIC: Autologous Matrix-induced Chondrogenesis, MFx: Microfracture, sp: Single plug, dp: Double plug, AOT: autologous osteochondral transplantation, CBMA: concentrated bone marrow aspirate | | | | | | | | |  |
