# Supplementary material for: Mid- to long-term outcomes of osteochondral lesions of the talus repair: a systematic review
Source: J Orthop Surg Res. 2025 Oct 14;20:892. doi: 10.1186/s13018-025-06214-z (PMC12522747; doi:10.1186/s13018-025-06214-z)
Supplement: Supplementary file 3 — Supplementary Material 3. [file 13018_2025_6214_MOESM3_ESM.docx]

**Table S2: Methodological Index for Non-Randomized Studies**

| **Author** | **Clearly stated aim** | **Inclusion of consecutive patients** | **Prospective collection of data** | **Endpoints appropriate to the aim of the study** | **Unbiased assessment of the study endpoint** | **Follow-up period appropriate to the aim of the study** | **Loss to follow up less than 5%** | **Prospective calculation of the study size** | **An adequate control group** | **Contemporary groups** | **Baseline equivalence of groups** | **Adequate statistical analyses** | **Total** | **Risk of Bias** |
| --- | --- | --- | --- | --- | --- | --- | --- | --- | --- | --- | --- | --- | --- | --- |
| **Anders 2012** | **2** | **2** | **2** | **2** | **1** | **2** | **2** | **1** | **-** | **-** | **-** | **-** | **14** | **Moderate** |
| **Baums 2006** | **2** | **1** | **2** | **2** | **2** | **2** | **2** | **1** | **-** | **-** | **-** | **-** | **14** | **Moderate** |
| **Becher 2019** | **2** | **2** | **2** | **2** | **1** | **2** | **2** | **2** | **0** | **0** | **2** | **2** | **19** | **Moderate** |
| **Becher 2015** | **2** | **2** | **2** | **2** | **2** | **2** | **2** | **2** | **-** | **-** | **-** | **-** | **16** | **Low** |
| **Berveglieri 2025** | **2** | **2** | **2** | **2** | **1** | **2** | **1** | **1** | **-** | **-** | **-** | **-** | **13** | **Moderate** |
| **Butler 2024** | **2** | **2** | **2** | **2** | **2** | **2** | **1** | **1** | **-** | **-** | **-** | **-** | **14** | **Moderate** |
| **Corr 2021** | **2** | **2** | **2** | **2** | **2** | **2** | **1** | **1** | **-** | **-** | **-** | **-** | **14** | **Moderate** |
| **Deiss 2024** | **2** | **2** | **2** | **2** | **2** | **2** | **1** | **1** | **-** | **-** | **-** | **-** | **14** | **Moderate** |
| **del'Escalopier 2021** | **2** | **1** | **2** | **2** | **1** | **2** | **1** | **2** | **-** | **-** | **-** | **-** | **13** | **Moderate** |
| **DiCave 2017** | **2** | **2** | **2** | **2** | **1** | **2** | **2** | **2** | **-** | **-** | **-** | **-** | **15** | **Low** |
| **Efrima 2024** | **2** | **2** | **2** | **2** | **1** | **2** | **2** | **2** | **-** | **-** | **-** | **-** | **15** | **Low** |
| **Fiske 2024** | **2** | **2** | **2** | **2** | **1** | **2** | **2** | **1** | **-** | **-** | **-** | **-** | **14** | **Moderate** |
| **Fu 2022** | **2** | **2** | **2** | **2** | **1** | **2** | **2** | **2** | **-** | **-** | **-** | **-** | **15** | **Low** |
| **Gedikbas 2024** | **2** | **2** | **2** | **2** | **2** | **2** | **1** | **2** | **0** | **0** | **2** | **2** | **19** | **Moderate** |
| **Giannini 2009** | **2** | **2** | **2** | **2** | **1** | **2** | **2** | **2** | **-** | **-** | **-** | **-** | **15** | **Low** |
| **Giannini 2014** | **2** | **0** | **2** | **2** | **1** | **2** | **2** | **2** | **-** | **-** | **-** | **-** | **13** | **Moderate** |
| **Gottschalk 2017** | **2** | **1** | **2** | **2** | **1** | **2** | **2** | **2** | **-** | **-** | **-** | **-** | **14** | **Moderate** |
| **Götze 2021** | **2** | **2** | **2** | **2** | **2** | **2** | **2** | **2** | **-** | **-** | **-** | **-** | **16** | **Low** |
| **Haleem 2014** | **2** | **2** | **2** | **2** | **1** | **2** | **2** | **2** | **0** | **0** | **2** | **2** | **19** | **Moderate** |
| **Keszég 2022** | **2** | **1** | **2** | **2** | **1** | **2** | **2** | **2** | **-** | **-** | **-** | **-** | **14** | **Moderate** |
| **Kim 2025** | **2** | **2** | **2** | **2** | **2** | **2** | **1** | **1** | **-** | **-** | **-** | **-** | **14** | **Moderate** |
| **Kreulen 2018** | **2** | **2** | **2** | **2** | **1** | **2** | **1** | **1** | **-** | **-** | **-** | **-** | **13** | **Moderate** |
| **Lambers 2021** | **2** | **0** | **2** | **2** | **1** | **2** | **1** | **2** | **-** | **-** | **-** | **-** | **12** | **Moderate** |
| **Lee 2025** | **2** | **2** | **2** | **2** | **2** | **2** | **2** | **1** | **2** | **2** | **1** | **1** | **21** | **Moderate** |
| **Lenz 2020** | **2** | **0** | **2** | **2** | **1** | **2** | **1** | **2** | **-** | **-** | **-** | **-** | **12** | **Moderate** |
| **Li 2023** | **2** | **2** | **2** | **2** | **1** | **2** | **2** | **2** | **-** | **-** | **-** | **-** | **15** | **Low** |
| **Manzi 2021** | **2** | **2** | **2** | **2** | **1** | **2** | **2** | **2** | **-** | **-** | **-** | **-** | **15** | **Low** |
| **Pagliazzi 2018** | **2** | **2** | **2** | **2** | **1** | **2** | **2** | **2** | **-** | **-** | **-** | **-** | **15** | **Low** |
| **Park 2021** | **2** | **2** | **2** | **2** | **2** | **2** | **1** | **2** | **-** | **-** | **-** | **-** | **15** | **Low** |
| **Polat 2016** | **2** | **2** | **2** | **2** | **1** | **2** | **2** | **2** | **-** | **-** | **-** | **-** | **15** | **Low** |
| **Richter 2019** | **2** | **0** | **2** | **2** | **1** | **2** | **2** | **1** | **-** | **-** | **-** | **-** | **12** | **Moderate** |
| **Richter 2022** | **2** | **2** | **2** | **2** | **1** | **2** | **1** | **2** | **-** | **-** | **-** | **-** | **14** | **Moderate** |
| **Rikken 2023** | **2** | **2** | **2** | **2** | **1** | **2** | **1** | **2** | **-** | **-** | **-** | **-** | **14** | **Moderate** |
| **Rikken 2024** | **2** | **2** | **2** | **2** | **2** | **2** | **1** | **2** | **-** | **-** | **-** | **-** | **15** | **Low** |
| **Shimozono 2019** | **2** | **2** | **2** | **2** | **2** | **2** | **2** | **2** | **0** | **0** | **2** | **2** | **20** | **Moderate** |
| **Suh 2024** | **2** | **2** | **2** | **2** | **1** | **2** | **1** | **1** | **-** | **-** | **-** | **-** | **13** | **Moderate** |
| **Toker 2020** | **2** | **2** | **2** | **2** | **1** | **2** | **2** | **2** | **-** | **-** | **-** | **-** | **15** | **Low** |
| **vanBergen 2013** | **2** | **1** | **2** | **2** | **2** | **2** | **2** | **2** | **-** | **-** | **-** | **-** | **15** | **Low** |
| **vanEekeren 2016** | **2** | **1** | **2** | **2** | **1** | **2** | **2** | **1** | **-** | **-** | **-** | **-** | **13** | **Moderate** |
| **Vannini 2023** | **2** | **2** | **2** | **2** | **1** | **2** | **2** | **2** | **-** | **-** | **-** | **-** | **15** | **Low** |
| **Viglione 2024** | **2** | **2** | **2** | **2** | **1** | **2** | **1** | **1** | **-** | **-** | **-** | **-** | **13** | **Moderate** |
| **Winkler 2023** | **2** | **2** | **2** | **2** | **2** | **2** | **2** | **2** | **-** | **-** | **-** | **-** | **16** | **Low** |
| **Yang 2025** | **2** | **2** | **2** | **2** | **1** | **2** | **1** | **2** | **0** | **0** | **2** | **2** | **18** | **High** |
| Green = 2 (reported and adequate), Orange = 1 (reported but inadequate), Red = 0 (not reported) | | | | | | | | | | | | | | |
