# Supplementary material for: Mid- to long-term outcomes of osteochondral lesions of the talus repair: a systematic review
Source: J Orthop Surg Res. 2025 Oct 14;20:892. doi: 10.1186/s13018-025-06214-z (PMC12522747; doi:10.1186/s13018-025-06214-z)
Supplement: Supplementary file 4 — Supplementary Material 4. [file 13018_2025_6214_MOESM4_ESM.docx]

**Table S3: Patient-Reported Outcomes**

| Joint preservation procedure | Author | Outcome Measurement | Preoperative PRO | Postoperative PRO | P value |
| --- | --- | --- | --- | --- | --- |
| Autologous chondrocyte implantation/ transplantations | Baums 2006 | Hannover ankle rating score | 40.4 (32 - 54) | 85.5 (64 - 100) | <0.001 |
|  |  | AOFAS | 43.5 ± 5.5 (37 - 57) | 88.4 ± 8.5 (66 -100) | <0.001 |
|  | Giannini 2009 | AOFAS | 89.4 ±14.5 | 92.7  ± 9.9 | <0.0005 |
|  | Giannini 2014 | AOFAS | 57.2 ± 14.3 | 92.0 ± 11.2 | 0.0005 |
|  | Pagliazzi 2018 | AOFAS | 58.7 ± 15.7 | 90.9 ± 12.7 | 0.0005 |
|  | Toker 2020 | AOFAS | 60.4±7.4 | 86.2±9.2 | ≤0.05 |
|  |  | VAS | 63±4 | 20±14 | NR |
|  | Viglione 2024 | AOFAS | 40.4 ± 19.8 | 94.7 ± 6.4 | < 0.0005 |
|  |  | NRS Pain | 78 ± 7 | 48 ± 21 | < 0.0005 |
|  |  | Tegner | 1 (1–3) | 3 (2–4) | < 0.0005 |
|  | Winkler 2023 | FAOS Symptoms | NR | 71.0 ± 24.5 | NR |
|  |  | FAOS Pain |  | 77.3 ± 21.0 |  |
|  |  | FAOS ADL |  | 83.3 ± 18.5 |  |
|  |  | FAOS sports/ recreation |  | 57.7 ± 34.3 |  |
|  |  | FAOS quality of life |  | 47.8 ± 28.6 |  |
|  |  | AOFAS |  | 89.6 ± 12.5 |  |
|  |  | VAS pain ankle |  | 31 ± 29 |  |
|  |  | Tegner activity scale |  | 3 |  |
|  |  | Lysholm score |  | 82.2 ± 21.6 |  |
|  |  | VAS pain knee |  | 9 ± 11 |  |
|  |  | MOCART |  | 73.7 ± 16.7 |  |
| Bone marrow Stimulation | Becher 2019 | HSS Microfracture | 58.4±11.2 | 82.9 ± 7.2 | <0.01 |
|  |  | HSS AMIC | 58.8±15.2 | 84.7±14.2 | <0.01 |
|  |  | VAS pain Microfracture | 89 ± 11 | 41 ± 25 | <0.01 |
|  |  | VAS pain AMIC | 87±21 | 33±23 | <0.01 |
|  |  | VAS function Microfracture | 37 ± 23 | 79±22 | <0.01 |
|  |  | VAS function AMIC | 49±26 | 85±21 | <0.01 |
|  |  | VAS satisfaction Microfracture | 34 ±24 | 79±24 | <0.01 |
|  |  | VAS satisfaction AMIC | 25±22 | 84±24 | <0.01 |
|  | Becher 2015 | Modified HSS | NR | 87 ± 12 | NR |
|  |  | AOFAS |  | 90 ± 13 |  |
|  |  | MOCART |  | 64 ± 14 |  |
|  | Corr 2021 | VAS pain | NR | 14 | NR |
|  |  | FAAM ADL |  | 90.29 |  |
|  |  | FAAM Sports |  | 82 |  |
|  | Polat 2016 | VAS | 79 ± 15 | 18 ± 7 | NR |
|  |  | AOFAS | 58.7 ± 5.2 | 85.5 ± 9.9 |  |
|  | Lambers 2021 | AAS | 6.2 | 5.8 | NR |
|  |  | NRS pain during rest | 27 | 10 | <0.01 |
|  |  | NRS pain during activity | 79 | 43 | <0.01 |
|  |  | FAOS Other | 64 | 52 | <0.01 |
|  |  | FAOS Pain | 61 | 67 | 0.01 |
|  |  | FAOS ADL | 66 | 79 | <0.01 |
|  |  | FAOS sport | 41 | 48 | <0.01 |
|  |  | FAOS QOL | 29 | 44 | <0.01 |
|  | Lee 2025 | FAOS pain | Smoker Group:64.9 ± 13.9  Non Smoker Group:62.9 ± 11.5 | Smoker Group: 84.3 ± 12.9  Non Smoker Group:84.8 ± 10.7 | N.S. |
|  |  | FAOS symptoms | Smoker Group:62.0 ± 14.7  Non Smoker Group:62.8 ± 14.0 | Smoker Group:85.2 ± 14.7  Non Smoker Group:82.8 ± 13.0 | N.S. |
|  |  | FAOS ADL | Smoker Group:75.2 ± 14.0  Non Smoker Group:75.1 ± 12.7 | Smoker Group:92.5 ± 8.4  Non Smoker Group:91.2 ± 8.0 | N.S. |
|  |  | FAOS Sport | Smoker Group:52.5 ± 15.2  Non Smoker Group:51.4 ± 15.2 | Smoker Group:78.7 ± 17.5  Non Smoker Group:77.9 ± 14.2 | N.S. |
|  |  | FAOS QOL | Smoker Group:47.2 ± 12.6  Non Smoker Group:46.6 ± 13.5 | Smoker Group:70.9 ± 18.0  Non Smoker Group:70.8 ± 15.9 | N.S. |
|  |  | AOFAS ankle-hindfoot score | Smoker Group:73.0 ± 8.2  Non Smoker Group:72.2 ± 6.7 | Smoker Group: 88.3 ± 10.6  Non Smoker Group:90.8 ± 7.4 | Improvement Smoker vs non smoker: 0.024 |
|  |  | SF-36 PCS score | Smoker Group:59.7 ± 13.1  Non Smoker Group:57.0 ± 12.5 | Smoker Group:74.3 ± 11.8  Non Smoker Group:76.2 ± 13.7 | Improvement Smoker vs non smoker: 0.046 |
|  |  | VAS for pain | Smoker Group: 51 ± 16  Non Smoker Group: 53 ± 13 | Smoker Group: 22 ± 18  Non Smoker Group:18 ± 14 | Improvement Smoker vs non smoker: 0.012 |
|  | Park 2021 | VAS | 71 ± 17 | 20 ± 17 | <0.001 |
|  |  | AOFAS | 58.2 ± 13.6 | 82.8 ± 11.7 | <0.001 |
|  | Rikken 2024 | NR | NR | NR | NR |
|  | vanBergen 2013 | AOFAS (Median) | NR | 88 | NR |
|  |  | SF-36 Vitality |  | 71 ± 16 |  |
|  |  | SF-36 Emotional |  | 94 ± 22 |  |
|  | vanEekeren 2016 | AAS | 8 (3-10) | 4 (2-10) | <0.001 |
|  | Yang 2025 | VAS | 77 ± 10 | 22 ± 18 | < 0.01 |
|  |  | AOFAS | 52.8 ± 21.2 | 88.0 ± 8.6 | < 0.01 |
|  |  | AAS | 1.9 ± 1.3 | 3.2 ± 0.7 | < 0.01 |
|  |  | FAOS symptom | 69.5 ± 22.4 | 80.3 ± 15.3 | < 0.01 |
|  |  | FAOS Pain | 58.8 ± 15.8 | 84.6 ± 10.2 | < 0.01 |
|  |  | FAOS Activities of Daily Living | 66.5 ± 18.7 | 92.5 ± 7.4 | < 0.01 |
|  |  | FAOS Sports | 53.3 ± 23.9 | 73.5 ± 16.9 | < 0.01 |
|  |  | FAOS QOL | 34.9 ± 12.1 | 62.7 ± 24.7 | < 0.01 |
| MACI | Anders 2012 | AOFAS | 70.1±8.3 | 95.3±5.6 | <0.001 |
|  |  | VAS | 57±26 | 09±8 | <0.001 |
|  |  | VAS subjective functional status | 53±23 | 89±9 | <0.001 |
|  |  | Tegner activity level | 2.4±1.2 | 4.7±0.6 | <0.001 |
|  |  | MOCART | 62.6 ±19.4 | 83.8±9.4 | NR |
|  | Kreulen 2018 | AOFAS | 61.8±14.3 | 78.3 ± 18.1 | 0.05 |
|  |  | SF-36 Physical | NR | Improvement | <0.01 |
|  |  | SF-36 Social |  | Improvement | <0.001 |
|  |  | SF-36 Pain |  | Improvement | <0.1 |
|  | Lenz 2020 | AOFAS | 60 ± 15 | 84±8 | <0.005 |
|  |  | FAAM ADL | NR | 89±11 | NR |
|  |  | FAAM LoF |  | 83±17 |  |
|  |  | VAS |  | 19±17 |  |
|  |  | MOCART |  | 65±23 (25-100) |  |
| OATS | Butler 2024 (Autograft) | FAOS | 51.9 ± 16.0 | 75.3 ± 21.9 | < 0.001 |
|  |  | VAS | 66 ± 18 | 16 ± 16 | < 0.001 |
|  | del'Escalopier 2021 (Autograft) | AOFAS | NR | 80.6± 19.4 | NR |
|  |  | FAAS |  | 77.8 ± 21.5 |  |
|  | Fiske 2024 (Fresh Allograft) | OMAS | 61.91 (57.61-66.21) | 84.37 (78.87-89.86) | NR |
|  |  | FAAM | NR | 68.91 (59.71-78.11) |  |
|  |  | FAAM Sports subscale |  | 70.96 (59.13-82.78) |  |
|  | Gedikbas 2024 (Autograft) | AOFAS | 47.7 ± 9.3 | 90.1 ± 4.1 | <0.001 |
|  |  | Freiburg ankle index | 46.3 ± 9.7 | 87.2 ± 6 | <0.001 |
|  |  | Tegner | 2.3 ± 0.6 | 5.4 ± 0.7 | <0.001 |
|  |  | VAS | 74 ± 9 | 31 ± 10 | <0.001 |
|  | Haleem 2014 (Autograft) | FAOS (Single Plug) | 51.6 ±  10.2 | 87.1 ± 5.1 | <0.001 |
|  |  | FAOS (Double Plug) | 49.5 ± 12.1 | 86.2 ±6.5 | <0.001 |
|  |  | SF-12 (Single Plug) | 57.8 ± 9.6 | 87.9 ±5.7 | <0.001 |
|  |  | SF-12 (Double plug) | 56.6 ±13.3 | 85.6 ±5.7 | <0.001 |
|  | Keszég 2022 (Autograft) | Tegner | 7.7 ± 1.1 | 6.3 ± 1.5 | <0.001 |
|  |  | FAOS | NR | 86.2 ± 16.1 | NR |
|  |  | MOCART |  | 50.8 ± 12.6 |  |
|  | Kim 2025 (Autograft) | FAOS total | 39.6±14.9 | 91.2±8.7 | <.001 |
|  |  | FAOS Pain | 33.2±16.5 | 90.3±9.5 | <.001 |
|  |  | FAOS Other symptoms | 49.4±18.1 | 93.5±7.2 | <.001 |
|  |  | FAOS Activities of daily living | 45.2±14.5 | 91.6±8.3 | <.001 |
|  |  | FAOS Sports and leisure | 25.8±13.3 | 87.9±10.2 | <.001 |
|  |  | FAOS Quality of life | 44.5±15.6 | 92.8±7.6 | <.001 |
|  |  | FAAM Total | 37.8±15.6 | 89.8±9.6 | <.001 |
|  |  | FAAM Daily Activty | 41.4±16.2 | 92.5±7.4 | <.001 |
|  |  | FAAM Sports Activity | 34.2±17.4 | 87.1±11.2 | <.001 |
|  | Shimozono 2019 (Autograft) | FAOS | 52.3 ± 16.7 | 75.5 ± 16.6 | <0.001 |
|  |  | FAOS with CBMA | 50.4 ± 15.5 | 80.5 ± 12.8 |  |
|  |  | SF-12 | 40.7 ± 18.0 | 69.6 ± 16.8 | <0.001 |
|  |  | SF-12 with CBMA | 40.1 ± 15.0 | 71.1 ± 17.1 |  |
|  | Suh 2024 (Autograft) | AOFAS | 55.4 ± 9.0 | 92.1 ± 7.6 | 0.001 |
|  |  | VAS | 55 ± 7 | 19 ± 8 | 0.001 |
|  |  | FAOS | NR | 89.1 ± 4.9 | NR |
| AMIC | Deiss 2024 | FFI-D | 56 ± 19 | 15 ± 13 | <0.001 |
|  |  | EFAS | NR | 18 ± 4 | NR |
|  |  | EFAS Sport |  | 11 ± 4 |  |
|  | Efrima 2024 | VAS during Walking (Median) | 70 (60-80) | 20 (0-30) | < 0.001 |
|  |  | AOFAS (Median) | 46.5 (38-64) | 92 (85-100) | < 0.001 |
|  |  | SF-12 physical (Median) | 31 (27-37) | 53 (45-56) | < 0.001 |
|  |  | SF-12 mental (Median) | 43 (40-46) | 56 (44-56) | < 0.001 |
|  |  | UCLA (Median) | 4 (3-6.5) | 7 (6-9) | < 0.001 |
|  |  | Halasi (Median) | 3 (3-5) | 4 (3-5) | 0.7 |
|  | Gedikbas 2024 | AOFAS | 53.3 ± 9.7 | 90.4 ± 4.8 | <0.001 |
|  |  | Freiburg ankle index | 55.2 ± 6.2 | 89.2 ± 7.3 | <0.001 |
|  |  | Tegner | 2.5 ± 0.8 | 5.7 ± 0.9 | <0.001 |
|  |  | VAS | 75 ± 9 | 30 ± 11 | <0.001 |
|  | Gottschalk 2017 | FFI-D | 56 ± 18 | 24 ± 21 | 0.457 |
|  |  | FFI-D Function | 58 ± 18 | 27 ± 23 | 0.482 |
|  |  | FFI-D Pain | 53 ± 21 | 21 ± 20 | 0.514 |
|  |  | MOCART | NR | 54 ± 14 | NR |
|  | Götze 2021 | FFI | NR | -1.3% | 0.8 |
|  |  | AOFAS |  | +3.4% | 0.2 |
| Biphasic Bioresorbable Scaffold | DiCave 2017 | AOFAS | 47.2 ± 10.7 | 84.4 ± 8 | <0.05 |
|  |  | VAS | 69 ± 14 | 12 ± 11 | <0.05 |
|  |  | MOCART | NR | 61.1 (25-85) | NR |
| MFx plus platelet-rich plasma and hyaluronic acid | Fu 2022 | AOFAS | NR | 85.1 ± 6.8 | NR |
|  |  | FAOS |  | 75.1 ± 9.0 |  |
|  |  | MOCART |  | 69.6 ± 10.5 |  |
| Autologous tibial osteoperiosteal grafts | Li 2023 | VAS | 63±  18 | 17  ±15 | <0.001 |
|  |  | AOFAS | 56.6  ±14.8 | 82.8  ±11.7 | <0.001 |
|  |  | SF-36 | 61.3  ±9.9 | 83.3  ±8.5 | <0.001 |
|  |  | MOCART | NR | 82.6 ± 8.4 | NR |
|  | Yang 2025 | VAS | 79 ± 13 | 15 ± 14 | <0.01 |
|  |  | AOFAS | 49.1 ± 19.9 | 88.7 ± 9.5 | <0.01 |
|  |  | AAS | 2.0 ± 1.0 | 3.8 ± 1.2 | <0.01 |
|  |  | FAOS symptom | 61.0 ± 24.1 | 86.5 ± 13.0 | <0.01 |
|  |  | FAOS Pain | 52.7 ± 17.3 | 90.4 ± 10.7 | <0.01 |
|  |  | FAOS Activities of Daily Living | 58.5 ± 19.2 | 93.1 ± 7.9 | <0.01 |
|  |  | FAOS Sports | 35.2 ± 25.7 | 75.5 ± 25.0 | <0.01 |
|  |  | FAOS QOL | 25.8 ± 15.1 | 72.7 ± 27.3 | <0.01 |
| Juvenile Particulated Cartilage Allograft Transplantation | Manzi 2021 | VAS | 68 ± 22 | 19 ± 16 | NR |
|  |  | AOFAS | 55.2 ±14.9 | 80.3 ± 16.3 |  |
|  |  | FAAM ADL | 46.5 ± 15.8 | 80.9 ± 16.4 |  |
|  |  | FAAM Sports | 18.8 ± 13.8 | 57.9 ± 28.3 |  |
|  |  | SF-36 Physical | 33.4 ± 7.8 | 78.9 ± 21.4 |  |
|  |  | SF-36 Mental | 55.2 ± 19.6 | 81.2 ± 13.2 |  |
| Matrix-associated stem cell transplantation | Richter 2019 | VAS FA | 45.2 | 84.4 | NR |
| Autologous matrix induced chondrogenesis plus peripheral blood concentrate | Richter 2022 | VAS FA | 45.7 | 84.2 | NR |
|  |  | EFAS | 9.8 | 21.5 |  |
| Arthroscopic Lift-Drill-Fill-Fix technique | Rikken 2023 | NRS pain at rest | 25 | 0 | <0.001 |
|  |  | NRS pain while walking | 70 | 0 | <0.001 |
|  |  | NRS pain while running | 80 | 20 | <0.001 |
|  |  | FAOS symptoms | 69.5 | 71.4 | Not Significant |
|  |  | FAOS pain | 66.5 | 94.4 | <0.01 |
|  |  | FAOS ADL | 90.5 | 98.5 | 0.02 |
|  |  | FAOS Sport | 40 | 80 | 0.01 |
|  |  | FAOS QoL | 22 | 56.3 | 0.02 |
|  |  | SF-36 physical | 43.3 | 45.1 | Not Significant |
|  |  | SF-36 Mental | 57.1 | 37.4 | <0.01 |
| Bone marrow aspirate concentrate scaffold | Vannini 2023 | AOFAS | 59.6 ± 13.9 | 82.3 ± 14.2 | <0.0005 |
|  |  | NRS Pain | 70 ± 13 | 39 ± 27 | <0.0005 |
|  |  | Tegner | 2.0 ± 1.5 (1-7) | 3.0 ± 1.5 (1-7) | <0.0005 |
|  | Berveglieri 2025 | AOFAS | 58.2 ± 14.1 | 85.17 ± 12.07 | < 0.05 |
|  |  | NRS | 7.5 ± 1.2 | 2.9 ± 2.6 | < 0.05 |
|  |  | Tegner | 4.7 ± 2.1 | 3.8 ± 1.71 | < 0.05 |
|  |  | EQ-5D Overall | 9.2 ± 1.3 | 5.7 ± 1.2 | < 0.05 |
|  |  | EQ-5D Mobility | 2.18 ± 0.5 | 1.21 ± 0.4 | < 0.05 |
|  |  | EQ-5D Care | 1.09 ± 0.3 | 1.01 ± 0.1 | < 0.05 |
|  |  | EQ-5D Activities | 2.24 ± 0.5 | 1.21 ± 0.4 | < 0.05 |
|  |  | EQ-5D Pain | 2.58 ± 0.5 | 1.27 ± 0.5 | < 0.05 |
|  |  | EQ-5D Anxiety | 1.09 ± 0.3 | 1.03 ± 0.2 | < 0.05 |
| AOFAS: American Orthopaedic Foot & Ankle Society score, VAS: Visual Analogue Scale, FAOS: Foot and Ankle Outcome Score, ADL: Activities of Daily Living, MOCART: magnetic resonance observation of cartilage repair tissue, HSS: Hannover Scoring System, CBMA: Concentrated Bone Marrow Aspirate, FAAM: Foot and Ankle Ability Measure, NRS: Numeric Rating Scale, QOL: Quality of Life, SF-36: Short Form Health Survey, FFI-D: Foot Function Index-Disability, VAS FA: Visual-Analogue-Scale Foot and Ankle, EFAS: European Foot and Ankle Society score, EQ-5D: EuroQol-5D, UCLA: University of California, Los Angeles score, OMAS: Olerud-Molander Ankle Score, N.S: Not significant | | | | | |
