# Supplementary material for: Mid- to long-term outcomes of osteochondral lesions of the talus repair: a systematic review
Source: J Orthop Surg Res. 2025 Oct 14;20:892. doi: 10.1186/s13018-025-06214-z (PMC12522747; doi:10.1186/s13018-025-06214-z)
Supplement: Supplementary file 5 — Supplementary Material 5. [file 13018_2025_6214_MOESM5_ESM.docx]

| Technique | Risk of Bias | Inconsistency | Indirectness | Imprecision | Publication Bias | GRADE Certainty |
| --- | --- | --- | --- | --- | --- | --- |
| Cell-Based | Serious | Not Serious | Serious | Serious | Undetected | Low |
| Scaffold-Based | Serious | Serious | Very Serious | Very Serious | Undetected | Very Low |
| Bone Marrow Stimulation | Serious | Not Serious | Serious | Serious | Undetected | Low |
| Osteochondral Grafting | Serious | Not Serious | Serious | Serious | Undetected | Low |

Table S4: GRADE Certainty
