# Supplementary material for: Mid- to long-term outcomes of osteochondral lesions of the talus repair: a systematic review
Source: J Orthop Surg Res. 2025 Oct 14;20:892. doi: 10.1186/s13018-025-06214-z (PMC12522747; doi:10.1186/s13018-025-06214-z)
Supplement: Supplementary file 6 — Supplementary Material 6. [file 13018_2025_6214_MOESM6_ESM.docx]

**Table S5: Rates of Complication, Revisions, and Survival Rates**

| Joint preservation procedure | Author | Complication | Revisions/ Reoperations | Survival Rate (%) | Returned to Activity/Sports | Level of Sport | Considerations |  |
| --- | --- | --- | --- | --- | --- | --- | --- | --- |
| ACI | Baums 2006 | None | None | 100 | Sports: 12/12 (100%) | 6 (50%) competitive, 6 (50%) light sports | None |  |
|  |  |  |  |  |  |  |  |  |
|  | Giannini 2009 | None | None | 100 | NR | | |  |
|  | Giannini 2014 | None | Failures revised with bone-marrow-derived cell transplantation (3) | 70 | Sports: 25/29 (86.2%) | 25 Recreational, 4 professional    16 contact sports  13 non-contact sports | 20/29 (69.0%) resumed sports at the same level, 3 (10.3%) resumed at a lower level, 2 (6.9%) shifted to non-contact sport, 4 (13.8%) gave up sports.    Four professional soccer players had best clinical and functional results, and were all able to resume sports |  |
|  | Pagliazzi 2018 | None | None | 100 | NR | | |  |
|  | Toker 2020 | Knee pain (3) | Second look arthroscopy for anterior ankle impingement (1) and pain (2), revision surgery (2) | 75 | NR | | |  |
|  | Viglione 2024 | NR | NR | NR | NR | | |  |
|  | Winkler 2023 | NR | NR | NR | Sports:  6/35 (17.1%) No limitation  8/35 (22.9%) minor limitation  21/35 (60%) major limitation  Work:  31/35 (88.6%) | NR | None |  |
| BMS | Becher 2019 | NR | NR | NR | NR | | |  |
|  | Becher 2015 | None | Arthroscopic microfracture for contralateral  osteochondral defect (1) | 93.3 | NR | | |  |
|  | Corr 2021 | NR | revision procedures of patients who had undergone prior arthroscopic chondroplasties (7), additional surgery due to failure (3) | 93.3 | Sports: 31/42 (74%) | NR | 36 (85.7%) stated ankle was not prohibiting them from sports, 5 (11.9%) patients choosing not to participate or not being able to for other reasons. |  |
|  | Polat 2016 | Transient superficial peroneal nerve neuropraxia (3).  Superficial infection (1)  Sinus tract formation (1) | Revision Therapy (2) | 97.6 | NR | | |  |
|  | Lambers 2021 | NR | Reoperations due to osteoarthritis (1), posterior Impingement (2) | 95 | Sports: 54/60 (90%)  Work: 58/60 (96.7%) | 6 professional (10%), 29 competitive (48.3%), 25 (41.7%) recreational | 32  (53%) at pre-injury level, 20 (33%) not at pre-injury level due to ankle problems, 8 (13%) not playing because of reasons not from ankle |  |
|  | Lee 2025 | None | NR | NR | NR | | |  |
|  | Park 2021 | None | Repeat BMS with debridement (5), AOT (6), total ankle arthroplasty (1) | 94.1 | NR | | |  |
|  | Rikken 2024 | NR | Revisions (33) | 87.4 | NR | | |  |
|  | vanBergen 2013 | Hypoesthesia of  superficial peroneal nerve (3) and first dorsal web (1) | Repeat debridement and BMS (4) | 92 | Sports: 37/42 (88.1%)  Work: 46/49 (93.9%) | NR | 2 (4.8%) changed type of sport |  |
|  | vanEekeren 2016 | NR | Repeat arthroscopy (4), Ankle fusion (1) | 92.6 | Sports: 71/93 (76.3%) | NR | At the time of final follow-up, more patients did more low contact sports compared to pre-injury sports |  |
| MACI | Anders 2012 | Limitations involved in dorsal extension of less than ten degrees (3), Temporary hypoaesthesia with regard to superficial nerve area (4) | None | 100 | Activity: 18/22 (81.8%) | NR | One patient (4.5%) exceeded preoperative level |  |
|  | Kreulen 2018 | None | Hardware removal (4) | 55.6 | NR | | |  |
|  | Lenz 2020 | NR | Revision for Soft tissue impingement (1) | 93.3 | NR | | |  |
| OATS | Butler 2024 | Excessive scar tissue formation (10), Symptomatic hardware (4), Wound infection (2), Transient donor site knee pain (1) | Secondary surgical procedures (15) | 61.5 |  | | |  |
|  | del'Escalopier 2021 | Osteoarthritis (22), persistent patellar syndrome (11) | None | 100 | NR | | |  |
|  | Fiske 2024 | NR | Further surgery (6) | 82.4 | Sports: 18/34 (52.94%) | 4 Competitive sports | NR |  |
|  | Gedikbas 2024 | Superficial wound infections (2), Cannulated screw removal (3), Knee pain (4), Ankle pain and functional limitations (5) | Diagnostic arthroscopy (3) | 94 | NR | | |  |
|  | Haleem 2014 | Knee stiffness (2), Saphenous nerve hypoesthesia (1) | NR | NR | NR | | |  |
|  | Keszég 2022 | Pain (2), limited in motion (2) | NR | NR | Sports: 23/24 (95.8%) | 17 (70.8%) Recreational, 7 (29.2%) competitive | 16 returned to same level, 7 at a lower level, 1 did not return    16 that returned same level were recreational, 7 that returned at lower level were competitive |  |
|  | Kim 2025 | Wound problems (2), Superficial peroneal nerve injury (1), Donor site morbidity (1) | None | 100 | Sports: 23/28 (82.1%) | NR | NR |  |
|  | Shimozono 2019 | Infection (1), Knee stiffness (1) | Arthroscopic debridement for anterior ankle impingement (5), arthroplasty for ankle arthritis (1) | 88.2 | NR | | |  |
|  | Suh 2024 | None | Cannulated screw removal (4) | 63.6 | NR | | |  |
| AMIC | Deiss 2024 | NR | NR | NR | NR | | |  |
|  | Efrima 2024 | NR | Arthroscopic removal of hypertrophic tissue (1), Revision arthroscopic microfracture surgery (1), Deterioration (3) | 92.1 | NR | | |  |
|  | Gottschalk 2017 | NR | NR | NR | NR | | |  |
|  | Götze 2021 | NR | Failures (2), Further AMIC due to growing pain (1) | 84.2 | NR | | |  |
| Biphasic Bioresorbable Scaffold | DiCave 2017 | None | None | 100 | NR | NR | Average return to sports was 6 months. All patients who did sports activities preoperatively returned to sports postoperatively. |  |
|  |  |  |  |  |  |  |  |  |
|  |  |  |  |  |  |  |  |  |
| MFx plus platelet-rich plasma and hyaluronic acid | Fu 2022 | NR | Reoperation (32) | 91.2 | NR | | |  |
|  |  |  |  |  |  |  |  |  |
|  |  |  |  |  |  |  |  |  |
| Autologous tibial osteoperiosteal grafts | Li 2023 | NR | Second look arthroscopy (8), reoperation due to poor incision healing (2) | 86.7 | NR | | |  |
|  |  |  |  |  |  |  |  |  |
|  |  |  |  |  |  |  |  |  |
|  |  |  |  |  |  |  |  |  |
|  | Yang 2025 | NR | NR | NR | NR | | |  |
| Juvenile Cartilage Replacement | Manzi 2021 | Hypertrophy of allograft cartilage at 2 year followup (1) | Additional procedure at time of implantation (6), Second look arthroscopy due to persistent pain (1) | 53.8 | NR | | |  |
| Matrix-associated stem cell transplantation | Richter 2019 | None | Joint preserving ankle surgery (3) | 97.5 | NR | | |  |
| Autologous matrix induced chondrogenesis plus peripheral blood concentrate | Richter 2022 | NR | Joint preserving ankle surgery (3), revision (1) | 96.9 | NR | | |  |
|  |  |  |  |  |  |  |  |  |
| Lift-Drill-Fill-Fix | Rikken 2023 | None | Unrelated surgeries (2), anterior cruciate ligament reconstruction (1), bi-planar chevron osteotomy for a symptomatic hallux valgus (1), revision surgery (2) | 66.7 | NR | | |  |
|  |  |  |  |  |  |  |  |  |
|  |  |  |  |  |  |  |  |  |
|  |  |  |  |  |  |  |  |  |
|  |  |  |  |  |  |  |  |  |
|  |  |  |  |  |  |  |  |  |
|  |  |  |  |  |  |  |  |  |
|  |  |  |  |  |  |  |  |  |
|  |  |  |  |  |  |  |  |  |
|  |  |  |  |  |  |  |  |  |
| Bone marrow aspirate concentrate scaffold | Vannini 2023 | NR | Prosthetic ankle replacement (3), additional BMAC (2), failures (5) | 90.1 | Activity: 69/101 (68.3%) | NR | None |  |
|  |  |  |  |  |  |  |  |  |
|  |  |  |  |  |  |  |  |  |
|  | Berveglieri 2025 | NR | Prosthetic ankle replacement (4) | 95.3 | NR | | |  |
| ACI: Autologous Chondrocyte Implantation, BMS: Bone Marrow Stimulation MACI: Matrix-associated Autologous Chondrocyte Implantation, OATS: Osteochondral autologous/allogenic transplantation, AMIC: Autologous Matrix-induced Chondrogenesis, MFx: Microfracture, NR: Not reported | | | | | | | |  |
