# Supplementary material for: Mid- to long-term outcomes of osteochondral lesions of the talus repair: a systematic review
Source: J Orthop Surg Res. 2025 Oct 14;20:892. doi: 10.1186/s13018-025-06214-z (PMC12522747; doi:10.1186/s13018-025-06214-z)
Supplement: Supplementary file 7 — Supplementary Material 7. [file 13018_2025_6214_MOESM7_ESM.docx]

**Table S6: Return to Activity and Sports**

| Author | Joint Preservation Technique | Number of Patients | Returned to Activity/Sports | Failed to Return to Activity/Sports | Level of Sport | Considerations |
| --- | --- | --- | --- | --- | --- | --- |
| Anders 2012 | MACI | 22 | Activity: 18/22 (81.8%) | 4/22 (18.2%) | NR | One patient (4.5%) exceeded preoperative level |
| Baums 2006 | ACI | 12 | Sports: 12/12 (100%) | 0% | 6 (50%) competitive, 6 (50%) light sports | - |
| Corr 2021 | BMS | 45 | Sports: 31/42 (74%) | NR | NR | 36 (85.7%) stated ankle was not prohibiting them from sports, 5 (11.9%) patients choosing not to participate or not being able to for other reasons. |
| DiCave 2017 | Biphasic Bioresorbable Scaffold | 12 | NR | NR | NR | Average return to sports was 6 months. All patients who did sports activities preoperatively returned to sports postoperatively. |
| Fiske 2024 | OAT | 34 | Sports: 18/34 (52.94%) | 16/34 (47.06) | 4 Competitive sports | - |
| Gianni 2014 | ACI | 46 | Sports: 25/29 (86.2%) | 4/29 (13.8%) | 25 Recreational, 4 professional  16 contact sports  13 non-contact sports | 20/29 (69.0%) resumed sports at the same level, 3 (10.3%) resumed at a lower level, 2 (6.9%) shifted to non-contact sport, 4 (13.8%) gave up sports.  Four professional soccer players had best clinical and functional results, and were all able to resume sports |
| Keszég 2022 | OATS | 24 | Sports: 23/24 (95.8%) | 1/24 (4.2%) | 17 (70.8%) Recreational, 7 (29.2%) competitive | 16 returned to same level, 7 at a lower level, 1 did not return  16 that returned same level were recreational, 7 that returned at lower level were competitive |
| Kim 2025 | OAT | 28 | Sports: 23/28 (82.1%) | 5/28 (17.9%) | - | - |
| Lambers 2021 | BMS | 60 | Sports: 54/60 (90%)  Work: 58/60 (96.7%) | 6/60 (10%) | 6 professional (10%), 29 competitive (48.3%), 25 (41.7%) recreational | 32  (53%) at pre-injury level, 20 (33%) not at pre-injury level due to ankle problems, 8 (13%) not playing because of reasons not from ankle |
| vanBergen 2013 | BMS | 50 | Sports: 37/42 (88.1%)  Work: 46/49 (93.9%) | 5/42 (11.9%) | NR | 2 (4.8%) changed type of sport |
| vanEekeren 2016 | BMS | 93 | Sports: 71/93 (76.3%) | 22/93 (23.7%) | NR | At the time of final follow-up, more patients did more low contact sports compared to pre-injury sports |
| Vannini 2023 | BMAC | 101 | Activity: 69/101 (68.3%) | 31/101 (31.7%) | NR | None |
| Winkler 2023 | ACI | 35 | Sports:  6/35 (17.1%) No limitation  8/35 (22.9%) minor limitation  21/35 (60%) major limitation  Work:  31/35 (88.6%) | NR | NR | None |
| MACI: matrix-associated Autologous Chondrocyte Implantation, ACI:Autologous Chondrocyte Implantation, BMS: Bone Marrow Stimulation, OATS: Osteochondral autologous/allogenic transplantation, BMAC: bone marrow aspirate concentrate scaffold | | | | | | |
